# Stress-Dependent Coordination of Transcriptome and Translatome in Yeast

**DOI:** 10.1371/journal.pbio.1000105

**Published:** 2009-05-05

**Authors:** Regula E Halbeisen, André P Gerber

**Affiliations:** Institute of Pharmaceutical Sciences, Department of Chemistry and Applied Biosciences, ETH Zurich, Zurich, Switzerland; University College London, United Kingdom

## Abstract

Cells rapidly alter gene expression in response to environmental stimuli such as nutrients, hormones, and drugs. During the imposed “remodeling” of gene expression, changes in the levels of particular mRNAs do not necessarily correlate with those of the encoded proteins, which could in part rely on the differential recruitment of mRNAs to translating ribosomes. To systematically address this issue, we have established an approach to rapidly access the translational status of each mRNA in the yeast Saccharomyces cerevisiae by affinity purification of endogenously formed ribosomes and the analysis of associated mRNAs with DNA microarrays. Using this method, we compared changes in total mRNA levels (transcriptome) with ribosome associations (translatome) after the application of different conditions of cellular stress. Severe stresses, induced by amino acid depletion or osmotic shock, stimulated highly correlated responses affecting about 15% of both total RNA levels and translatome. Many of the regulated messages code for functionally related proteins, thus reflecting logical responses to the particular stress. In contrast, mild stress provoked by addition of Calcofluor-white and menadione altered the translatome of approximately 1% of messages with only marginal effects on total mRNA, suggesting largely uncorrelated responses of transcriptome and translatome. Among these putative translationally regulated messages were most components of the mitochondrial ATPase. Increased polysome associations of corresponding messages and higher mitochondrial ATPase activities upon treatment confirmed the relevance for regulation of this macromolecular complex. Our results suggest the presence of highly sensitive translational regulatory networks that coordinate functionally related messages. These networks are preferentially activated for rapid adaptation of cells to minor environmental perturbations.

## Author Summary

Organisms respond to environmental or physiological changes by altering the amounts and activities of specific proteins that are necessary for their adaptation and survival. Importantly, protein levels can be modulated by changing either the rate of synthesis or the stability of the messenger RNA (mRNA or transcript), or the synthesis or stability of the protein itself. Scientists often measure global mRNA levels upon changing conditions to identify transcripts that are differentially regulated, and often the assumption is made that changes in transcript levels lead to corresponding changes in protein levels. Here, we systematically compared global transcript levels (transcriptome) with global alterations in the levels of ribosome association of transcripts (translatome) when yeast cells are exposed to different stresses to determine how significant the discrepancy between transcript and protein levels can be. We found that changes in the transcriptome correlate well with those in the translatome after application of harsh stresses that arrest cell growth. However, this correlation is generally lost under more mild stresses that do not affect cell growth. In this case, remodeling of gene expression is mainly executed at the translational level by modulating mRNA association with ribosomes. As one example, we show that expression for many components of the mitochondrial ATPase, the major energy production machinery in cells, is translationally but not transcriptionally activated under a specific mild stress condition. Our results therefore show that alteration of protein synthesis can be the dominant mediator of changes of gene expression during adaptation to minor changes in cellular needs.

## Abstract 3

During cellular adaptation to changing growth conditions, the extent of correlation between changes in transcriptional and translational regulation varies with the severity of the stress.

## Introduction

Gene expression is regulated at diverse levels to achieve coordinate synthesis of the cell's macromolecular components. Besides transcriptional regulation, it has become increasingly evident that gene expression is controlled by a network of highly interconnected posttranscriptional regulatory factors, such as RNA-binding proteins and noncoding RNAs [1–4]. Consistently, the posttranscriptional regulation of protein synthesis plays essential roles for development, oncogenesis, and synaptic plasticity [5–7].

Translation is thought to be mainly controlled at the initiation step where eukaryotic initiation factors (eIFs) recruit the small ribosomal subunit (40S subunit) and scan the 5′-untranslated region (UTR) of the mRNA for the start codon. The initiation factors are then released, and the large ribosomal subunit (60S) joins the complex to form a fully assembled, translationally competent ribosome. Modification of initiation factors, such as phosphorylation of eIF2α, prevents formation of the initiation complex and thus globally represses translation initiation of most messages. Likewise, the availability of initiation factors, such as eIF4e, is controlled by 4E-binding proteins that inhibit association of the 40S subunit with the mRNA [8]. In yeast, the depletion of nutrients triggers such global repression within minutes, manifested by a gradual decrease of polysomes for most transcripts. The accumulating “pool” of mRNAs is largely incorporated into so-called processing bodies (P-bodies) where they are degraded or kept translationally silent [9]. Besides global repression of translation, more specific modes of regulation can be observed for individual messages. For instance, translation of *GCN4* is activated in response to amino acid deprivation—a condition that generally represses translation—by a mechanism that involves short upstream open reading frames (uORFs) [10]. In addition, translation of specific messages can also be controlled by specific RNA-binding proteins (RBPs) [8,11]. Intriguingly, many regulatory RBPs interact with functionally related groups of mRNAs, also referred to as “posttranscriptional operons,” suggesting highly coordinated control at all steps of posttranscriptional gene regulation [2,12–14].

Several genome-scale studies have been undertaken to decipher the extent of translational regulation upon diverse stimuli in eukaryotes [1,15]. Classical sucrose density fractionation has been commonly applied to separate “free” RNA and monosomes from polysomes, followed by a systematic analysis of the relative distribution of mRNAs in these two fractions using DNA microarrays. In most cases, the global response on transcript levels was quantified in parallel to disclose the relation between nascent transcription/RNA turnover and translation [16–20]. For several stress conditions such as amino acid deprivation, high salinity, and heat shock, the imposed remodeling of translation often reflects a magnification of the changes of transcript levels. This effect has been termed “potentiation,” and may in part be explained by the deposition of regulatory proteins on certain mRNAs during transcription [18,21]. However, under other stress conditions, such as butanol or peroxide addition, these coordinate reactions could not be observed [19,20]. Clearly, details about the extent and regulation of potentiation remain to be resolved. Nevertheless, the specific sets of mRNAs that underwent treatment specific regulation shared some common functional theme that can be attributed to a logical response of the cell to its altered physiological circumstances.

The above-mentioned studies exposed cells to “severe” stress conditions, triggering global repression of translation and cell growth. However, it is well established that under these conditions, yeast cells respond in a stress-specific manner but also by a common program that changes the steady-state level of hundreds of messages in a stereotypic way, commonly referred to as the environmental stress response (ESR) [22]. ESR includes approximately 900 genes; 600 genes are commonly repressed and mainly encode proteins for growth-related processes including ribosomal proteins, whereas 300 genes are up-regulated and refer to proteins acting in carbohydrate metabolism, intracellular signaling, or stress, such as chaperones and DNA damage repair enzymes. In contrast, little is known about the number and nature of messages that undergo translational regulation under “mild” stress conditions, i.e., stimuli that affect cellular homeostasis without triggering ESR. Such minor changes could reflect adaptive responses that may occur very frequently in a cell.

To measure possible small-scale adaptive responses following the application of mild stress, a highly sensitive and precise method of accessing a cell's translatome has to be employed. To this end, we have established a method based on the purification of affinity-tagged ribosomes followed by analysis of associated mRNAs with DNA microarrays. The method allows rapid access to purified ribosomes devoid of contaminations by lipid rafts, processing body (P-body) components, or pseudo-polysomes, which may be present in classical polysomal gradients [23]. We have then globally analyzed reactions of global transcript levels (transcriptome), which are influenced by nascent transcription and RNA decay, and the translatome upon the application of different stress conditions. Severe stress, imposed by amino acid deprivation and addition of sorbitol prompting an osmotic shock, revealed highly coordinate programs that may largely rely on potentiation. In contrast, mild stress, which was applied by the addition of noninhibitory concentrations of Calcofluor-white (CFW), a cell-wall–perturbing agent, and menadione, an inducer of mild oxidative stress, leads to a noncorrelated response preferentially changing the translatome profiles. Taken together, our data suggest a model in which the relocation or translational regulation of existing mRNA “pools” primarily takes place to achieve rapid adaptation to changing environmental conditions.

## Results

### Affinity Purification of Ribosomes

Epitope-tagged proteins have been used for affinity purification of ribonucleoprotein (RNP) complexes followed by the identification of protein and RNA components with mass spectrometry and genomics tools, respectively [12,13,24–26]. We aimed to adapt this approach for the rapid purification of yeast ribosomes and the analysis of associated mRNAs with DNA microarrays. Previously, Inada et al. [27] used FLAG-(His)_6_-tagged ribosomal protein L25 (Rpl25p) to capture monosomes and polyribosomes (polysomes) from yeast extracts with an anti-FLAG agarose affinity resin. Although ribosomal proteins, ribosomal RNAs (rRNAs), and mRNAs were successfully copurified with the Rpl25p bait, large polysomes greater than five ribosomes were underrepresented [27]. Thus, their strategy may not completely reflect the translational status of mRNAs. However, in a more recent study in plants, the careful selection of the tagged ribosomal protein appeared to circumvent this problem so that larger polysomes could also be purified [28].

**Figure 1:**
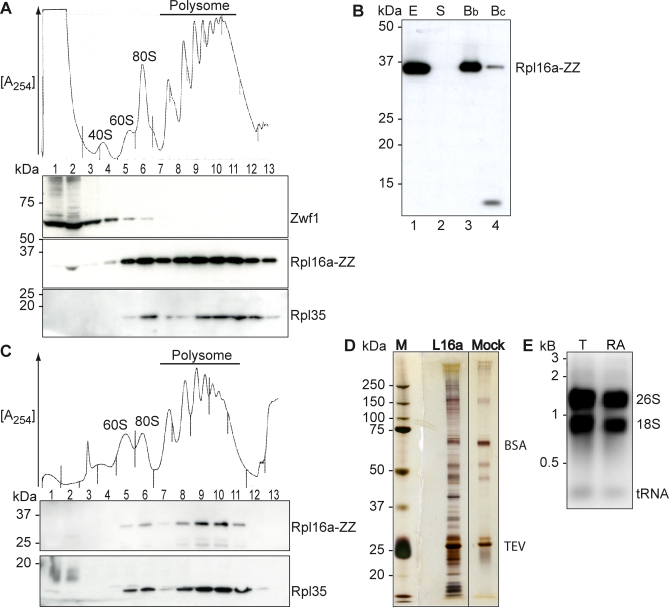
Affinity Purification of Tagged Ribosomes (A) Polysomal association of tagged Rpl16a protein in cells grown in YPD medium. The absorbance profile at 254 nm of a sucrose gradient is shown at the top, and the positions of the 40S and 60S ribosomal subunits, 80S monosomes, and polyribosomes are indicated. Fractions were separated by SDS-PAGE, blotted, and probed with Peroxidase-Anti-Peroxidase Soluble Complex (PAP) to detect tagged Rpl16p (Rpl16a-ZZ), and with specific antibodies to detect Rpl35p (a ribosomal protein) and Zwf1p (a cytoplasmic protein not associated with polysomes). Molecular masses are indicated in kilodaltons on the left. (B) Immunoblot analysis following the affinity purification. Lanes: 1, cell-free extract (E); 2, supernatant after incubation of extracts with IgG beads (S); 3, captured beads (Bb); and 4, beads after elution with TEV protease (Bc). (C) Polysomal profile of affinity-purified ribosomes isolated from cells grown in SC medium. (D) Affinity-purified ribosomes (Rpl16a) were separated by SDS-PAGE, and proteins were visualized with silver (L16a). A sample from a control purification with untagged wild-type cells is also shown (Mock). Bands representing BSA and TEV protease are marked, and the protein marker (M) with molecular masses is depicted to the left. (E) Total RNA isolated from extracts (T), and from affinity-purified ribosomes (RA) was separated on a 1% denaturing agarose gel, and RNA was visualized with ethidium bromide. The positions of 26S, 18S ribosomal RNAs, and tRNAs are indicated to the right. Lengths are indicated in kilobases (kb) on the left.

Therefore, we decided to use tagged versions of solvent-exposed ribosomal proteins for the affinity purification of ribosomal particles. To achieve stable expression of each protein under its endogenous promoter, we integrated the tags at the original chromosomal location by homologous recombination, more specifically, at the C-terminus of the corresponding open reading frame (ORF). Initially, we used cells expressing tandem affinity purification (TAP)-tagged Rpl25. However, these cells exhibited severe growth defects when cultured in rich media (unpublished data). We then tested another solvent-exposed ribosomal protein, Rpl16, a C-terminally tagged version of which was previously shown to be efficiently incorporated into polysomes [29,30]. In the S. cerevisiae genome, two paralogous copies of the *RPL16* gene exist, termed *RPL16A* and *RPL16B*, and we generated ZZ-tagged versions of either gene. The ZZ-tag, which comprises part of the TAP-tag [26], contains two protein A immunoglobulin G (IgG)-binding units and a tobacco-etch virus (TEV) protease recognition sequence allowing elution of the bound material from the affinity resin [12,31,32]. Both the *rpl16a-ZZ* and the *rpl16b-ZZ* strain showed the same growth behavior and cell morphologies as wild-type cells. In addition, we found that *rpl16a* and *rpl16b* deletion strains showed no differential growth behavior under the stress conditions described below (Figure S1, and unpublished data). Sucrose density gradient (10% to 50% w/v) centrifugation revealed that both paralogous proteins were well incorporated into actively translating polysomes (Figure 1A; and unpublished data). Tagged ribosomes were recovered from cell lysates by affinity selection on IgG-coupled beads and subsequently released from the matrix by cleavage TEV protease. However, using standard IgG-coupled agarose beads, we could only recover approximately 10%–20% of the tagged ribosomes from extracts, with large polysomes being underrepresented in the purified fraction, reminiscent of the results obtained by Inada et al. [27] with FLAG-tagged Rpl25 (unpublished data). Increasing the amount of beads relative to extract did not significantly increase ribosome recovery. Agarose-IgG is a porous gel support, with an exclusion limit of 20,000 kDa for a 4% solution (http://www.piercenet.com). This could be refractory for the binding of polysomes with more than five ribosomes (4,200 kDa each) [33]. To overcome this limitation, we tested IgG-coupled, spherical microbeads with 1-μm diameter. The diameter of a ribosome is 50 times smaller (∼20 nm), so these beads should allow efficient recovery of large polysomes with no sterical hindrance. Indeed, using microbeads, we recovered more than 95% of tagged ribosomes from extracts (Figure 1B). Polysomal profiles of purified ribosomes revealed the presence of 60S subunits, monosomes, and polysomes, including large polysomes, whereas free 40S subunits were absent (Figure 1C). These results show that spherical microbeads are superior to porous agarose beads for the efficient recovery of large RNP complexes such as polysomes.

The protein and RNA content of affinity-purified ribosomes was visualized on protein and RNA gels, respectively (Figure 1D and 1E). From 100-ml yeast cultures, we obtained approximately 45 μg of total RNA, including 26S and 18S rRNA. In contrast, less than 1 μg of total RNA was isolated from untagged wild-type control cells (mock control). Moreover, corresponding protein gel analysis revealed only major bands for TEV protease and BSA, the blocking reagent used during the procedure. To assess the purity of isolated ribosomes, we further analyzed eluates from Rpl16a-ZZ–captured ribosomes by liquid chromatography–tandem mass spectrometry (LC-MS/MS). We identified 31 of the 78 ribosomal proteins (40%), including the known ribosome-associated proteins Asc1p and Yhr113p [34–36] (for a complete list of identified proteins by mass spectrometry [MS], see Table S1). Likely, we were not able to identify the remaining 37 ribosomal proteins because of the limited resolution of our MS analysis since our polysomal profiles of the affinity-purified material indicated the recovery of fully assembled ribosomes, including Rpl35p, a protein that was not identified by MS (Figure 1C). Nevertheless, we found two proteins not expected to be associated with ribosomes: the ATPase Ino80p, which is part of a chromatin remodeling complex that contains actin and several actin-related proteins located at stalled replication forks, and which may promote the resumption of replication upon recovery from fork arrest [37]; and Rrn7p, which is part of the core factor complex that is involved in the regulation of Pol I–mediated transcription of 35S rRNA genes [38,39]. Whether these nuclear proteins have additional functions on ribosomes remains to be elucidated. In conclusion, these results suggest that we specifically and efficiently purified complete and translationally competent ribosomes with only marginal unspecific binding. Therefore, we reason that the pool of mRNAs associated with the purified ribosomes should represent the translatome, which we define as the fraction of all messages localized to ribosomes prone to translation.

### Analysis of Ribosome-Associated mRNAs

We analyzed the RNA content of purified ribosomes (translatome) isolated from cells grown in minimal medium using oligo microarrays. To achieve this, total RNA isolated from extracts of cells expressing Rpl16a-ZZ or Rpl16b-ZZ (input) and from the affinity-purified ribosomes was reverse transcribed with oligo(dT) primers. cDNA was labeled with Cy3 and Cy5 fluorescent dyes, respectively, and competitively hybridized to yeast oligo microarrays. In this assay, the ratio of the two RNA populations at a given array element reflects the relative enrichment of the respective mRNA with ribosomes, i.e., highly enriched features represent transcripts that are highly associated with ribosomes, whereas underrepresented features are only marginally associated with ribosomes, as is expected for nuclear RNAs, mitochondrial encoded genes, or messages that are stored in other cytoplasmic RNP pools such as P-bodies. The enrichment (log_2_ ratios) from two independent biological replicates was averaged and ranked (a complete dataset is presented in the supporting information; Dataset S1). We found that the overall enrichment of mRNAs associated with Rpl16a and Rpl16b correlated fairly well (Pearson correlation = 0.73; Figure S2) and hence was similar to the correlations seen between independent biological replicates (*r* = 0.7). The data was narrowly distributed (standard deviation of log_2_ ratios = 0.53), and only 324 (5.2%) and 277 (3.8%) of all expressed messages exhibited a more than 2-fold difference from the mean of all analyzed features with Rpl16a-ZZ and Rpl16b-ZZ, respectively (Figure S2). As expected, mitochondrial encoded transcripts (average log_2_ ratio = −1.7/−2.9 with Rpl16a/b) and introns (average log_2_ ratio = −0.95) were among the least enriched RNAs. The lowest-ranked nuclear encoded mRNA was *HAC1* (average log_2_ ratio = −1.8), a well-known translationally regulated message that has been reported to be associated relatively weakly with ribosomes [40]. Similarly, *ICY2* mRNA, which is also translationally regulated [16], was among the least-enriched messages (average log_2_ ratio = −1.1). Therefore, we propose that the 106 mRNAs that were found to be more than 2-fold negatively enriched (*p* ≤ 0.05) in either Rpl16a or Rpl16b affinity isolates may constitute messages that undergo translational regulation (for a list of these candidates, see Dataset S1). A total of 260 (4.2%) and 127 (1.8%) features were more than 2-fold enriched in affinity isolates of Rpl16a-ZZ and Rpl16b-ZZ, respectively. Some of them code for proteins related to metabolism, such as pyruvate metabolism (Rpl16a: ten genes, *p* < 7 × 10^−6^), alcohol biosynthesis (Rpl16a: 11 genes, *p* < 3 × 10^−5^), and glucose catabolism (Rpl16a: seven genes, *p* < 0.003), probably reflecting the high metabolic activity of cells grown in minimal media. Interestingly, Ty elements were also highly associated with purified ribosomes (average log_2_ ratios = 1.2 with Rpl16a) (Figure S2), which is contrary to their relatively weak association in cells grown in rich media (unpublished data), and may be indicative of the activation of transposition events to create advantageous genetic changes for the cell's adaptation to stress [41,42].

The good correlation of Rpl16a- and Rpl16b-associated mRNA profiles (Figure S2), and the above-mentioned equal behavior of *rpl16a* and *rpl16b* mutant cells under diverse stress conditions (Figure S1) suggests that both paralogs associate with mRNAs to similar efficiencies without a detectable bias for a certain subset of messages. This finding is important in light of the recent observation that paralogous ribosomal proteins may differentially regulate specific messages under certain conditions [43]. Although both paralogs behaved similarly, we have decided to use Rpl16a-ZZ for further experiments.

To compare Rpl16-associated mRNAs with the respective enrichments in polysomes, we also performed classical sucrose density fractionation experiments. We isolated RNA from the 60S, 80S, and polysomal fractions, which correspond to the fractions represented in Rpl16 affinity isolates (Figure 1C). As described for the analysis of Rpl16 affinity isolates, we then determined the relative enrichment of mRNAs in these fractions by competitive hybridization with total RNA samples (Dataset S1). The data obtained from four independent replicates similarly correlated as replicates of Rpl16a or Rpl16b affinity isolations (*r* = 0.5–0.7; for a list of Pearson correlations, see Dataset S1). *HAC1* mRNA, introns, and other noncoding RNAs were among the least enriched messages compared to the global transcript levels, whereas many mRNAs coding for ribosomal proteins were among the most enriched. We found that mRNA enrichment in polysomes (normalized log_2_ ratios) weakly to fairly correlated with the enrichment profiles obtained from Rpl16a or Rpl16b affinity-purified ribosomes (*r* = 0.3–0.5). The reasons for this fair correlation could be manifold and should be interpreted with care. On the one hand, the polysomal fractions may be contaminated with other high molecular weight complexes that are not an integral part of ribosomes such as lipid rafts, P-body components, or pseudo-polysomes [23,34]. These “contaminants” could add messages that are not primarily associated with ribosomes, and hence, this may impinge on the microarray profile. On the other hand, the different experimental procedures and RNA extraction protocols may affect the composition of the respective RNA pools. For instance, separation of ribosomal components by sucrose density fractionation leads to rather diluted RNA samples compared to the relatively concentrated samples obtained from ribosome affinity isolates. Moreover, the application of high centrifugal force combined with the relatively long experimental procedure to collect sucrose density fractions may potentially lead to some dissociation of ribosomes from messages or to partial RNA degradation.

### Stress-Type–Dependent Correlation of Global Transcript Levels and Translatome

To analyze how changes in steady-state mRNA levels are related to changes of respective messages in the translatome, total RNA and ribosome-associated RNA from stress-treated and untreated cells were analyzed with yeast cDNA microarrays. To achieve this, fluorescently labeled (Cy5) cDNA was prepared from total RNA isolated from yeast cell extracts and from affinity-purified ribosomes. Each sample was then mixed with differentially labeled (Cy3) cDNA prepared from a common reference RNA pool, and competitively hybridized on yeast cDNA microarrays. We performed five independent experiments with untreated cells (*t* = 0 reference) and three or more independent biological replicates of treated cells (Dataset S2). Altogether, we sampled the cell's response to five different stress treatments that were applied for relatively short periods of time (10 or 20 min) to avoid secondary effects triggered by transcriptional adaption [22]. We applied two distinct classes of stress: severe stress, which leads to cell growth inhibition and ESR, and mild stress, which does not affect cellular growth. Severe stress should reveal general, possibly well-coordinated responses of gene expression as previously seen for amino acid starvation [20], salinity response [17], heat shock, and rapamycin treatment [18]. We provoked this type of stress by removing amino acids for 10 and 20 min, or by initiating osmotic shock with 1 M sorbitol for 10 min. To monitor the effect of these treatments on translation, we recorded polysomal profiles from treated cells (Figure S3). Amino acid starvation induced major translational arrest already by 10 min, with an increased monosome fraction and fewer polysomes compared to untreated control cells. In contrast, treatment of cells with 1 M sorbitol for 10 min only marginally changed polysomal profiles, consistent with recent findings that hyperosmotic force created by the addition of 1 M NaCl changes the sedimentation profile of polysomal complexes slowly, with maximum reduction of polysomes after 1 h [17]. Mild stress was provoked by the application of two different concentrations of CFW, an antifungal agent that binds to chitin with high affinity and prevents normal cell-wall assembly [44]. At low doses (10 μg/ml), it does not cause a discernable phenotype, whereas 10-fold higher doses (100 μg/ml) induce the formation of cell clumps, which are probably caused by repeated budding without completing the separation of mother and daughter cells [44]. We also applied 1 mM menadione to cells [22], which induces mild oxidative stress by producing a flux of superoxide anions in the cell [45]. The application of CFW and menadione for 20 min did not alter cell growth or polysomal profiles, suggesting that translation was not globally repressed (Figure S3, and unpublished data).

Under all severe stress conditions, the relative changes of mRNA levels, which may be the result of altered transcription and/or RNA decay, correlated well with those of ribosome associations (translatome) (Pearson correlation *r* = 0.75–0.81; scatter plots depicting the changes in global transcript levels and translatome are shown in Figure S4). This indicates a surprisingly high degree of correlation in the response of global transcript levels and translatome even after short periods of severe stress. In contrast, we observed no, or only weak, correlation of global transcript levels and the translatome after mild stress treatments (*r* = 0.01–0.56). In this case, changes of relative expression were preferentially found for ribosome-associated messages (translatome) with only minor changes at global transcript levels, which is indicative for preferential remodeling of the translatome upon weak environmental perturbations. Notably, the low degree of correlation is not simply due to limited applicability of the arrays for measuring minute changes, since mRNA expression and translatome profiles correlated well across independent biological replicates (*r* > 0.6).

**Figure 2:**
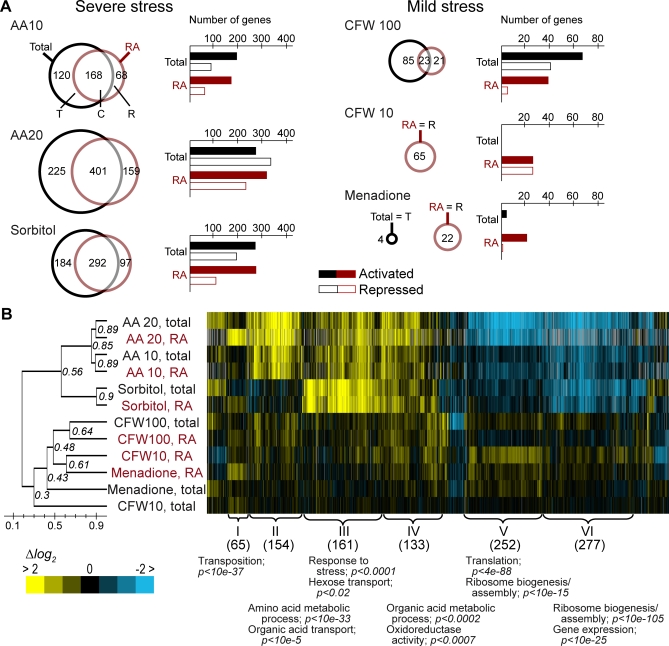
Stress-Specific Transcriptome and Translatome Profiles (A) Venn diagrams showing the overlap of genes that significantly changed steady-state mRNA levels (Total, black circle) and ribosome associations (RA, red circle) after application of the indicated stress. C: set of genes that was significantly changed in both groups; R: set of genes with preferentially altered ribosome associations (translatome); T: set of genes with significantly altered mRNA levels (more than 2-fold, *p* ≤ 0.05) but no significant change of ribosome association. The bars to the right refer to the number of up- or down-regulated genes. (B) A hierarchically clustered heat map of transcriptome and translatome profiles of stress-treated cells. The cluster contains 1,422 genes that changed more than 2-fold (*p* ≤ 0.05) in at least one of the stress conditions as specified in (A). Each row represents an experiment set (averaged from independent replicates), and each column represents one gene. Pearson correlations of experiments are indicated and visualized with a tree. The relative expression of genes is represented with a blue–yellow color bar, where blue means lower and yellow means higher expression compared to untreated cells. Subclusters are numbered with roman letters, and the number of genes and significantly enriched GO terms are indicated.

To generate a list of significantly changed genes upon each stress treatment, we arbitrarily selected those messages that changed more than 2-fold with a *p*-value of less than 0.05 in either global transcript levels or translatome (Figure 2A). To visualize the relation among genes and experiments, we hierarchically clustered the 1,422 selected genes (Figure 2B; for a list of selected messages, see Dataset S3) [46]. Severe stress caused significant changes in approximately 20% of global transcript levels and translatome (1,305 genes), whereas mild stress affected only a much smaller (206 genes, 3% of all messages) and largely distinct subset of genes, including merely 89 messages that were changed in both classes of treatments (Figure 2B). Moreover, the close coordination of global transcript levels and translatome under severe stress conditions is largely lost under mild stress conditions (CFW, 10 μg/ml, and menadione), as reflected by the lower correlation of the respective transcriptome and translatome profiles (Figure 2B). A more detailed discussion of the gene cluster is provided in the Text S1.

**Figure 3:**
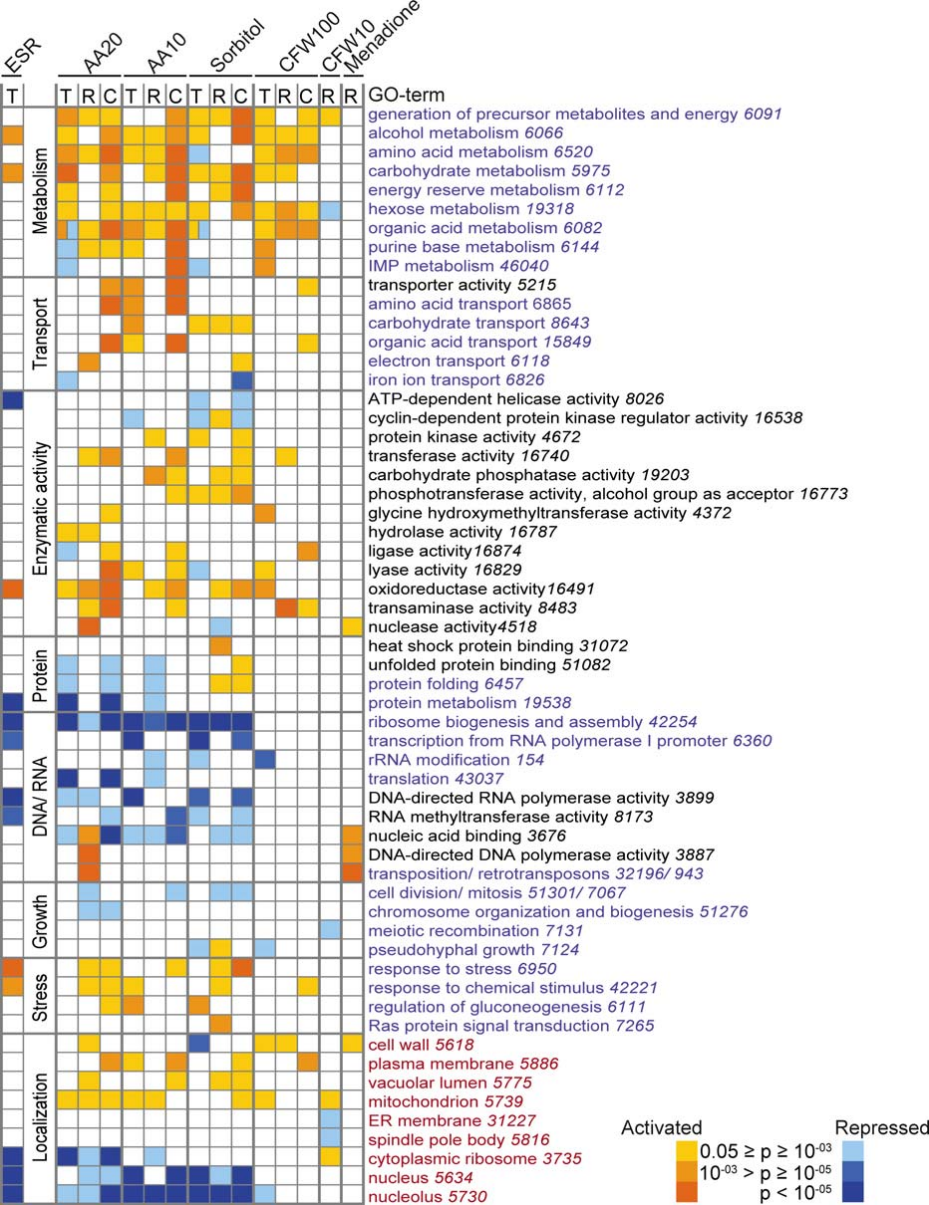
Enrichment of Functional Themes among Stress-Regulated Messages Enrichment of GO terms among significantly changed gene sets (T, C, and R) specified in the upper-left Venn diagram shown in Figure 2A. The significance of enrichment of the GO term is represented as a heat map in which the color code corresponds to *p*-values (scale is indicated at the bottom). Themes among up-regulated genes are shown in yellow–red scale, themes among down-regulation genes are in blue scale. GO terms are colored according to their classification: “biological process” in blue, “biological function” in black, and “cellular component” in red; GO identification numbers are added in italics. Enrichment of respective GO terms among the ESR genes [22] is shown to the left.

We systematically searched for shared Gene Ontology (GO) annotations among the messages with altered steady-state levels (transcriptome) or ribosome association (translatome), as well as those that changed concomitantly (Figure 3). The latter set comprises genes for which changes in steady-state mRNA levels and translation are relatively strong and well correlated, whereas the less well-coordinated group of genes may represent candidates for further posttranscriptional regulation, e.g., by regulated translation or mRNA decay. In the following section, we briefly describe the groups of messages with changed mRNA steady-state levels or ribosome association under each specific stress condition.

### Coherent and Coordinate Responses of Global Transcript Levels and Translatome upon Severe Stress

Relative changes of the global transcript levels upon amino acid starvation are in good agreement with previously published data (*r* = 0.7) [22], with 5% (288 genes after 10 min) and 10% (626 genes after 20 min) of transcripts altering more than 2-fold (*p* ≤ 0.05). Likewise, 4% (236 genes, 10 min) and 9% (560 genes, 20 min) of messages had significantly altered ribosome associations (Figure 2A). Most of the genes overlapped among these subsets, and 97% of mRNAs showed homodirectional changes in ribosome association and transcript levels, substantiating the notion for highly correlated responses of nascent transcription/RNA turnover and the translatome. To a large extent, the proteins encoded by these mRNAs can be grouped into common functional themes such as amino acid biosynthesis, energy generation, and transport, for which relative abundances were generally increased, whereas messages coding for proteins acting in ribosome biogenesis and translation were preferentially decreased (Figure 3). We hypothesize that these effects represent a coordinate and logic response to activate metabolic processes and transport of metabolites to substitute for missing compounds (amino acids), and to globally reduce ribosome biosynthesis and translation in order to slow down cell growth and save energy before and during adaptation to the new environment. However, we also found messages that are differentially changed at transcript levels and translatome: 68 mRNAs (1% of all genes analyzed) and 159 mRNAs (2.5%) were significantly changed in affinity isolates of ribosomes but did not change more than 2-fold in total RNA isolates of cell extracts after 10 and 20 min of amino acid starvation, respectively (Figure 2A). Among these, 42 (135) messages had increased ribosome association after 10 min (20 min) of amino acid depletion including the message of *GCN4*, a gene coding for a transcription factor known to be translationally regulated under these conditions [47]. To more selectively identify potential candidates for translational regulation, we further normalized the translatome to the transcript-level profiles by subtracting the relative changes of transcript levels from that of the translatome, and we selected those messages that were more than 2-fold (*p* ≤ 0.05) changed. (The number of selected messages and the GO analysis is provided in Figure S5). In the case of amino acid starvation for 20 min, only 78 messages (1% of expressed genes) were selected by this analysis, further reflecting the above-mentioned strong correlation of global transcript levels and translatome profiles. These messages preferentially encode nucleic acid- or protein-binding proteins, including *GCN4* (ΔΔlog_2_ ratio = 1.43). The selection of *GCN4* in both analyses corroborates the validity of our approach to identify translationally regulated messages. Whether other messages in the selected groups undergo specific translational regulation will require further investigation.

A total of 7.5% (476 genes) of global transcript levels and 6% (389 genes) of the translatome changed significantly in cells subjected to hyperosmotic stress (Figure 2A). Here, 96% of the messages with altered transcript levels showed homodirectional changes in ribosome association, concomitantly increasing relative mRNA levels and translation of mRNAs coding for proteins that function in carbohydrate metabolism and transport, as well as the generation of precursor metabolites and energy. In contrast, genes coding for proteins involved in ribosome biogenesis and assembly and those acting in the nucleus or nucleolus were generally repressed (Figure 3). Ninety-seven transcripts (1.5%) were significantly changed at the translatome, but not at the total RNA level; many of the transcripts encode proteins involved in carbohydrate metabolism (e.g., Hxk2, Mdh1; *p* < 0.003), transport (e.g., Hxt2, Mal31; *p* < 0.049), and stress response (e.g., Rrd1, Yim1; *p* < 0.018). This subset also contained the message for *SUT1*, which became strongly associated with ribosomes (>3-fold) but had slightly decreased steady-state mRNAs levels upon stress treatment (0.7-fold quantified by quantitative reverse-transcriptase (RT)-PCR. *SUT1* (YGL162w) encodes a transcription factor that regulates genes involved in sterol uptake. The transcript contains a short uORF [48,49]; uORFs have been shown to regulate the translation of the downstream coding sequence and the stability of the mRNA, further supporting that *SUT1* may be translationally regulated [50]. To validate our microarray data and to see whether *SUT1* messages become differentially associated with polysomes, we recorded the polysomal distribution of *SUT1* (YGL162W) mRNA before and after osmotic stress (Figure S6). Indeed, the amount of mRNA present in translation impotent fractions (1–5) was reduced by approximately 35%, providing further evidence that this message is subject to translational regulation (Figure S6).

### Preferential Response of Translatome upon Mild Stress

Whereas steady-state mRNA levels in cells treated with low doses of CFW (10 μg/ml) remained largely unchanged (average log_2_ ratio = 0.03), the ribosome association status of 65 mRNAs (∼1% of analyzed genes) altered significantly more than 2-fold (Figure 2A). Some of these mRNAs encode ion transporters or proteins involved in energy generation, including mitochondrial ATPase components (see below), which are known to mediate cell-wall stress response [51]. Moreover, whereas translation-related genes are generally repressed upon severe stress and ESR, a fraction of the respective messages, mainly coding for ribosomal proteins, became preferentially recruited to ribosomes (Figure 2B; cluster IV). This finding is also reflected by our second type of analysis selecting messages that are more than 2-fold differentially altered at translatome compared to global transcript levels, which revealed 22 messages coding for ribosomal proteins (*p* < 10^−5^; Figure S5). We speculate that cells may increase translation, through enhanced production of ribosomes, to replace damaged proteins at the cell periphery.

Interestingly, the treatment of cells with higher doses of CFW (100 μg/ml) induced a largely unrelated response compared to that of cells treated with the 10-fold lower dose affecting 1.5% (108 mRNAs) of the global transcript levels and 0.5% (44 mRNAs) of translatome (Figure 2). As seen with severely stressed cells, the changes of these selected messages were largely homodirectional (93%), possibly reflecting a potentiated response. Moreover, those mRNAs with increased levels belong to particular functional themes, mostly connected to intermediary metabolism, as seen for severely stressed cells (Figure 3). Contrariwise, the general repression of DNA/RNA-related processes in severely stressed cells was not observed with CFW, which is in agreement with unchanged polysomal profiles. Therefore, this treatment reflects an intermediate response that includes some characteristics of severely stressed cells.

As observed with low doses of CFW, mild oxidative stress induced by menadione preferentially altered the translatome in a reaction that does not correlate with respective changes in global transcript levels (Figure 2). Twenty of the 22 messages with more than 2-fold changed ribosome associations were activated, and several of them encode proteins with cell-wall regulatory functions, such as Ecm33 and Uth1, the latter of which has been reported to play a role in oxidative stress response [52]. We speculate that other messages in this group could also code for proteins that specifically protect cells from damage by oxidative stress, but this needs further investigation.

### Translational Activation of the Mitochondrial ATPase

**Figure 4:**
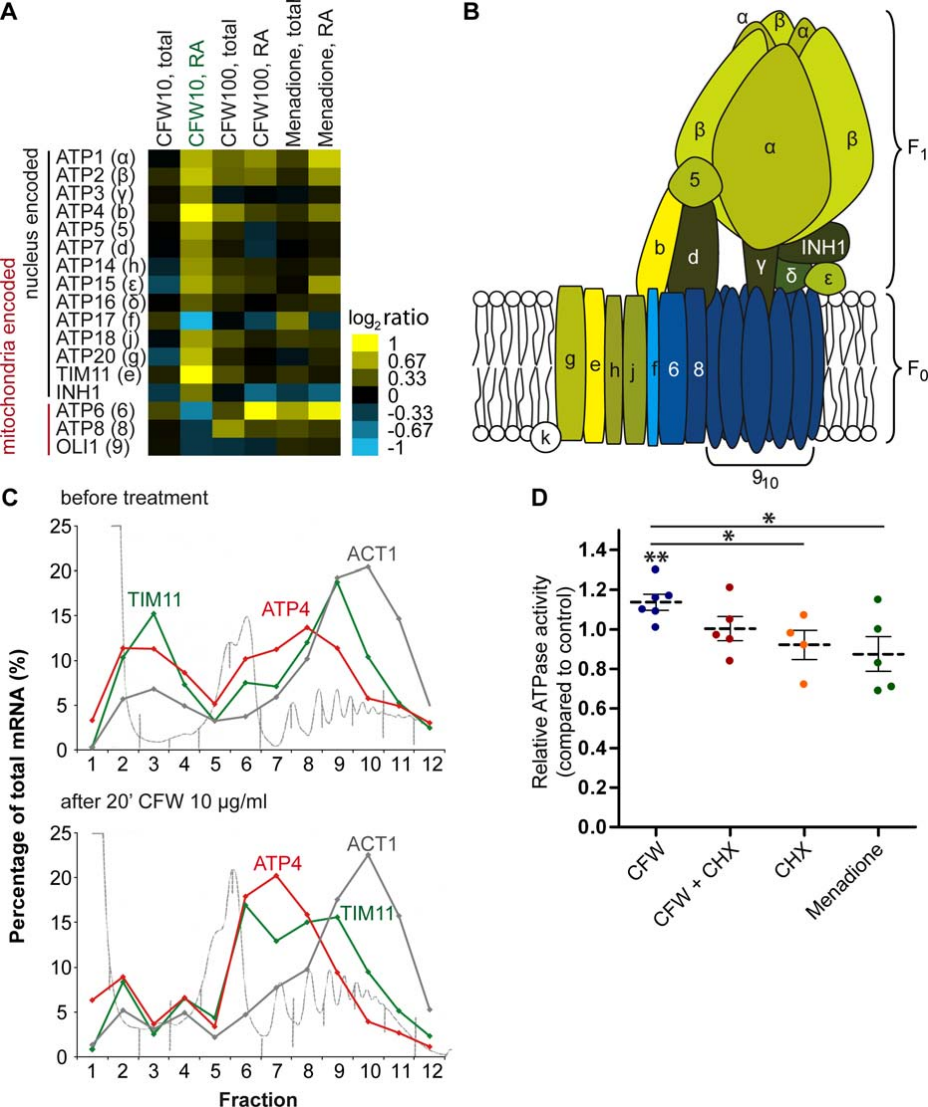
Enhanced Ribosome Association and Activity of the Mitochondrial F_1_F_0_-ATPase by Low Doses of CFW (A) Heat map representing relative changes of expression of 17 mitochondrial F_1_F_0_-ATPase components after mild stress treatments (blue–yellow color scale). Total: changes of steady-state mRNA levels, RA: change of ribosome associations. The gene names are indicated to the left, with the name of the corresponding subunit in parentheses. (B) Structure of the mitochondrial F_1_F_0_-ATPase. Each component is colored according to changes in ribosome associations depicted in (A). The membrane-associated part (F_0_) uses a proton motive force to mechanically drive the soluble part (F_1_) that exhibits ATPase activity [78,79]. (C) Distribution of TIM11, ATP4, and ACT1 mRNAs in polysomal gradients obtained from untreated cells (upper panel), and from cells treated with CFW (lower panel). RNA was isolated from each fraction of the polysomal profile and quantified by RT-qPCR (see Materials and Methods).The mRNA level in each fraction was calculated as a percentage of the total; data and absorbance (254 nm) profiles from representative experiments are plotted. ACT1 is as a negative control mRNA that was not expected to alter ribosome association. (D) Relative mitochondrial ATPase activity of drug-treated versus untreated control cells. Cells were treated with CFW (CFW) for 20 min; preincubated for 10 min with CHX prior to addition of CFW (CHX + CFW); or treated with CHX or menadione. The activity of purified mitochondria was normalized to the untreated control sample. Average activities are indicated with a dashed line, standard errors of the mean (SEM) with continuous lines. Single dots represent biologically independent experiments (double asterisks [**] indicate *p* < 0.01; a single asterisk [*] indicates *p* < 0.05).

We were intrigued by the finding that eight of the 14 nuclear-encoded messages (*p* < 0.002) coding for components of the mitochondrial ATPase showed at least 1.5-fold increased ribosome association after application of low doses of CFW (10 μg/ml), whereas total mRNA levels remained unchanged (Figure 4A and 4B). Moreover, this common up-regulation seems to be specific since it was not observed under the other tested stress conditions (Figure 4A). These data suggest that low doses of CFW specifically induce the “relocalization” of those mRNAs to ribosomes that code for the mitochondrial ATPase complex. To test this hypothesis, we monitored the distribution of ATP4 and TIM11 mRNAs, coding for two components of the complex, within polysomal profiles by quantitative RT-PCR (RT-qPCR) in untreated and CFW-treated cells (10 μg/ml) (Figure 4C). Both messages became increasingly associated with monosomes or polysomes in treated cells, reducing the fraction of non–ribosome-associated messages in fractions 1 to 5 by 27% (TIM11) and 38% (ATP4), respectively. Total mRNA levels were only marginally altered in treated versus untreated control cells as quantified by RT-qPCR (TIM11 = 1.1-fold; ATP4 = 1.2-fold; normalized to ACT1). We visualized the distribution of ACT1 mRNA as a control and found it unchanged in polysomal profiles upon treatment.

We next wondered whether the increased recruitment of mRNAs to ribosomes correlates with enhanced protein synthesis. However, since classical pulse-chase experiments to measure de novo protein synthesis rely on periods of methionine starvation that induce a stress response, we were reluctant to use this approach because it may simply overwhelm the relatively small effects of the mild CFW stress treatment. Instead, we thought to directly monitor enzymatic activity of the mitochondrial ATPase activity. Therefore, we isolated mitochondria from treated and untreated yeast cells, and determined ATPase activity in the presence or absence of oligomycin, a drug that specifically inhibits the mitochondrial ATPase [53] (Figure S7). We found that mitochondria isolated from CFW-treated (10 μg/ml) cells showed 14% increased activity compared to the ones isolated from solvent-only–treated control cells (*n* = 6, *p* = 0.006; Figure 4C). To distinguish whether the increased activity is dependent on protein synthesis or is the result of posttranslational regulation that simply alters activity of the preexisting complex, we preincubated the cells with the translational inhibitor cycloheximide (CHX) for 10 min prior to treatment with CFW. Under these conditions, we observed that the activity of the complex was not significantly changed compared to the solvent control (*n* = 5, *p* = 0.98). Likewise, treatment with CHX alone did not reveal altered activity compared to control samples (*n* = 5, *p* = 0.3). As an additional control, cells were treated with 1 mM menadione, and consistent with the corresponding array data, the ATPase activity was also not significantly changed (*n* = 5, *p* = 0.2). In conclusion, these results suggest that for nuclear-encoded components of the mitochondrial ATPase complex, low doses of the cell-wall–perturbing agent CFW specifically trigger the enhanced recruitment of their corresponding messages to ribosomes, leading to increased protein synthesis, and ultimately, higher ATPase activity. To our knowledge, this is the first example for translational regulation of a small macromolecular complex in eukaryotes.

## Discussion

We have established an approach to rapidly access the translatome of yeast cells. In this regard, the translatome refers to the pool of all RNAs that are associated with ribosomes purified via an affinity tag. Using this approach, we compared the relative changes of translatome and global transcript levels in response to five conditions of stress. This analysis provides a catalog of stress-regulated messages comprising approximately 20% of all expressed genes. Most of the messages changed homodirectionally, particularly under severe stress, suggesting a strong coordinate response of global transcript levels and translatome. Our survey suggests that only about 2% of all expressed messages were differentially regulated, preferentially under mild stress conditions and therefore represent candidates for translational regulation. Among these, we showed that the mitochondrial ATPase is prone to translational regulation in response to low doses of the cell-wall–perturbing agent CFW.

We accessed the translatome through the affinity purification of Rpl16, a tagged ribosomal protein of the 60S subunit, and subsequent analysis by microarrays (Figure 1). This is distinct from previous approaches aimed to study translational regulation, which applied “classical” sucrose density fractionation to separate mRNAs present in polysomes from “free” RNA and those messages present in monosomes [54]. Our alternative approach captures all messages that are bound by at least one ribosome (monosome) but excludes free RNA and those messages incorporated into other RNP complexes such as P-bodies or pseudo-polysomes, which may be present in classical polysomal gradients [9]. Possibly, this may also be a reason for the rather fair correlation (*r* = 0.3–0.5) seen between the relative enrichments of messages associated with tagged ribosomes and corresponding fractions from classical sucrose density profiles.

We have used affinity-purified ribosomes to assess the recruitment of individual messages to ribosomes on a genome-wide level, covering all steps of translation initiation, which is believed to be a major step for translational regulation [8]. We wish to note that we may not be able to monitor regulation by alterations of ribosome density or elongation rates. Such an analysis requires monitoring of mRNAs in polysomal profiles as we did for selected candidate messages (Figures 4 and S6). Instead, our approach allows several additional applications that cannot be realized with classical sucrose density gradients. For instance, it offers the possibility to study mRNAs associated with paralogous ribosomal protein. Although our data do not indicate preferential association of mRNAs with either tagged paralogous Rpl16a and Rpl16b proteins grown in minimal media (Dataset S1), the preferential association of selected messages with paralogs may become vital under certain specific growth conditions, especially in the light of recent observations that paralogous ribosomal proteins may be required for proper expression of certain messages [43]. Tagged ribosomal proteins may also be used to access tissue-specific expression in animals or in complex cell suspensions in vitro. Previously, affinity-tagged poly(A)-binding protein was expressed with tissue-specific promoters to identify muscle- or neuron-specific messages in nematode Caenorhabditis elegans or in the fruit fly Drosophila melanogaster and included cross-linking of the proteins to their bound RNAs with formamide [55,56]. The use of tagged ribosomal protein may represent a valuable alternative that could involve short treatment with CHX to stall ribosomes on messages. Indeed, while this manuscript was under revision, Heintz and colleagues reported the generation of transgenic mice expressing tagged Rpl10a to identify mRNA expressed in certain cell-types of mouse neurons [57,58].

Here, we used our approach to monitor responses of the translatome upon the application of diverse stress conditions and investigated how they relate to respective changes in the global transcript levels (transcriptome) (Figure 2). We found that severe stress, leading to growth arrest and ESR, induces highly balanced, correlated, and precise responses of transcript levels and translatome. This strong coordination even within minutes after stress application is intriguing, as it suggests that there is basically no delay of the regulating response of transcription and mRNA decay. In contrast, we found a largely uncorrelated response of global transcript levels and translatome under mild stress conditions: Low doses of CFW and menadione almost exclusively remodeled the translatome with very minor effects on the transcriptome. It appears that under these conditions, the cells adapt primarily at the posttranscriptional level, through the differential recruitment of messages to ribosomes and hence the regulation of protein synthesis. A possible explanation for this reaction could be that cells “buffer” environmental artifacts that do not greatly affect cell function or viability. Cells may favor keeping transcription constant because restructuring requires energy and additional organization.

Unfortunately, only a few studies have been performed that correlate protein levels with mRNA abundance, and to our knowledge, no systematic analysis of the changes of yeast protein levels upon stress is currently available [59,60]. In a recent study investigating mRNA levels and abundance of 450 proteins, it was demonstrated that 73% of the variance in protein abundance can be explained by mRNA abundance [60]. Conversely, this finding suggests that about one quarter of the variance of protein levels may be additionally controlled by translation or protein turnover. On the basis of our findings, we predict that only a minor fraction of this variance is due to altered ribosome recruitment. It may be possible that under steady-state conditions, the variance is mainly based on altered translation rates, or most likely, altered protein turnover. It will certainly be interesting to simultaneously measure alterations in transcriptome, translatome, and proteome to get a comprehensive view of the regulatory impact on each level of gene expression.

Not surprisingly, we observed that under severe stress conditions, many of the commonly or selectively altered messages in the transcriptome (global transcript levels) and translatome code for functionally related proteins; mRNAs coding for proteins that act in metabolism, transport, or are homed to the generation of energy showed increased relative abundances, whereas those involved in cell growth, protein or DNA/RNA binding and translation were preferentially decreased, many of them being part of the ESR [22] (Figure 3). The repression of components for the protein and mRNA synthesis machineries correlates with retarded cell growth, an effect that has been observed in various instances when cells adapted to new conditions [16,22,61]. On the other hand, we speculate that increased production of components acting at the cell periphery (plasma membrane, cell wall) may be dedicated to replace damaged proteins or to intensify sensing of the environment.

Even under mild stress conditions that only affected ribosome association of a minor fraction of messages (up to 1%), we found various examples for concerted themes among messages in the translatome. For instance, the increased ribosome association for most components of the mitochondrial ATPase, which was confirmed by respective shifts of selected messages in polysomal profiles and the CHX-dependent increase of the mitochondrial ATPase activity in treated cells, strongly suggests that this small macromolecular complex undergoes translational regulation. The mitochondrial ATPase provides the major energy source of cells [62]. Therefore, tight regulation of this complex at all levels of gene expression may be pivotal for proper energy homeostasis. There are no reports on how expression of this complex is achieved in yeast, but its biogenesis and assembly, in particular the uneven stochiometry of components for both F_1_ and F_0_ which are even encoded in different cellular compartments (Figure 4B), certainly requires highly coordinate regulation, possibly involving posttranscriptional control. In the green algae *Chlamydomonas*, for example, it has indeed been shown that formation of the chloroplast ATPase is dependent on controlled translation of subunits in the cytoplasm and in the chloroplast [63].

Besides *cis*-acting elements in the mRNAs that may directly affect translation rates of specific messages as seen for GCN4, translational regulation may also occur by *trans*-acting factors, such as specific RBPs and noncoding RNAs. Dozens of regulatory RBPs co-sediment with ribosomes and polysomes, and thus could potentially regulate translation of specific sets of mRNAs [34]. Intriguingly, we and others have shown that regulatory RBPs often bind to messages that encode functionally related proteins [2,11–14,64,65]. It is also striking that some RBPs preferentially bind to messages coding for proteins either at the cell periphery (e.g., cell wall) or the nucleus [14]. Coincidentally or not, we found that messages related to these themes were also preferentially regulated upon application of stress. Furthermore, our studies suggest that regulation of translation, in particular the recruitment of messages to ribosomes, may be a prominent mechanism to escape or balance slight environmental perturbations. Since it appears that such buffering reactions also involve functionally related groups of messages, and such functional coherent sets are often bound by RBPs, we speculate that RBPs may be significantly involved in this process. If so, the plethora of RBPs and their bound mRNAs define a magnitude of overlapping “posttranscriptional operons” and RNA regulons [1–4,66]. This may constitute the primary mediator of environmental responses that may even shape the cell's individual behavior.

## Materials and Methods

### Oligonucleotide primers.

The ZZ-tag was amplified with primers RPL16A-F2, 5′-CTACTGCTGCTGAATCTGATGTTGCTAAACAATTAGCCGCTTTGGGTTACcggatccccgggttaattaa-3′, and RPL16A-R1, 5′-TTGTGATTATATTACAAAATGTAAAGTATTAAAGAAAAACTATTTTTAATgaattcgagctcgtttaaac-3′. Genomic integration of the ZZ-tag was verified with primers annealing in the kanamycin-cassette (Kan-Rev; 5′-GTATTCTGGGCCTCCATGTCGC-3′) and the gene-specific primer RPL16A-Fc (5′-GCGTTTACGAGTCCCCAAGG-3′). The following primers were used for RT-qPCR: ACT1-Fw, 5′-ATGGTCGGTATGGGTCAAAA-3′; ACT1-Rv, 5′-TCCATATCGTCCCAGTTGGT-3′; SUT1-Fw, 5′-CCATTGCTTGGCCATTAAGT-3′; SUT1-Rv, 5′-ACATCTTTTGATGGCGGTTC-3′; TIM11-Fw, 5′-ATACTCTGCGTTGGGTTTGG-3′; TIM11-Rv, 5′-TGTGCCTGCTCCTCTTTCTT-3′; ATP4-Fw, 5′-GGTGTTCGTTGTCCTTCGAT-3′; ATP4-Rv, 5′-CCTGGAATGGCATTGATGAT-3′; and LYS1-Fw, 5′-CTGAAAGCACAGGTGGCATA-3′; LYS1-Rv, 5′-AGCTCTCTCCGGATACGACA-3′.

### Yeast, media, and stress treatments.

BY4741 (MATa *his3Δ0 leu2Δ0 met15Δ0 ura3Δ0*) wild-type cells or *rpl16* mutant cells derived from this strain [67] were cultured at 30 °C in synthetic complete dextrose (SC) or Yeast-Peptone-Dextrose (YPD) medium where explicitly mentioned [68]. Stress treatments were performed as follows: Cells were grown to mid-log phase (optical density at 600 nm [OD_600_] ∼0.5), collected by filtration and further grown in medium lacking amino acids (amino acid starvation) or supplemented with 1 M sorbitol for 10 min to induce an osmotic shock. Mild stress was induced by the addition of 10 or 100 μg/ml (final concentration) CFW (Sigma) or 1 mM menadione (Sigma) for 20 min. In all cases, 0.1 mg/ml CHX (Sigma) was added 1 min prior to harvesting cells to block translational elongation [40]. Cells were rapidly collected by vacuum filtration (HVPL type filters [0.45 μm]; Millipore), frozen in liquid nitrogen, and stored at −80 °C. Phenotypic growth studies were performed on SC plates supplemented with 10 or 100 μg/ml (final concentration) CFW (Sigma). Cells were plated in a series of 10-fold dilutions and grown for 3 d at 30 °C.

### Yeast extract preparation.

One hundred milliliter yeast cultures were grown to mid-log phase (OD_600_ = 0.5) and collected as described above. Cells were washed on filters with 10 ml of ice-cold buffer A (20 mM Tris-HCl [pH 8.0], 140 mM KCl, 2 mM MgCl_2_, 1% Triton X-100, 0.2 mg/ml heparin [Sigma], 0.1 mg/ml CHX) and flash-frozen in liquid nitrogen. After resuspension in 1 ml of buffer B (buffer A plus 0.5 mM dithiothreitol [DTT], 1 mM phenylmethylsulfonylfluoride [PMSF], 0.5 μg/ml Leupeptin, 0.2 μg/ml Pepstatin, 20 U/ml DNase I, 100 U/ml RNase Out [Invitrogen]) in a glass Corex tube, cells were broken mechanically with one-third volume of 0.5-mm glass beads by vortexing four times at full speed for 20 s followed by 90-s cooling steps on ice in between. Cell lysates were cleared by three subsequent centrifugation steps (2,600*g*, 8,600*g*, and 13,400*g*, 5 min, 4 °C) in a microcentrifuge and brought to 1 ml with buffer B.

### Immunoblot analysis.

Proteins were separated by SDS-polyacrylamide gel electrophoresis (PAGE) and transferred to nitrocellulose membranes (BioRad) with a semidry electrophoretic transfer cell (Trans Blot SD, BioRad). Membranes were blocked in phosphate-buffered saline–0.05% Tween-20 (PBST) containing 5% low fat milk, probed for 1 h at room temperature (RT) with designated antibodies and horse radish peroxidase (HRP)-coupled secondary antibodies, and developed with the ECL Plus Western Blotting Detection System (Amersham). The following antibodies were applied (dilution indicated in parentheses): peroxidase anti-peroxidase soluble complex (PAP) reagent (Sigma; 1:5,000) for detection of the ZZ-tag, rabbit anti-Rps3p (1:100,000), rabbit anti-Rpl35p (1:20,000) [69], rabbit anti-Zwf1p (Sigma, 1:5,000) and mouse anti-Por1p (Invitrogen, 1:2,500). Bands on membranes were quantified with Quantity One (BioRad) software.

### Sucrose density fractionation.

A total of 0.8 ml of extract was loaded on top of a 10% to 50% sucrose gradient prepared in buffer B without Triton X-100. The samples were centrifuged in a Beckman SW-41 rotor for 160 min at 35,000 rpm and 4 °C, and then fractionated while continuously recording the absorbance at 254 nm (A_254_) with a flow cell UV detector (ISCO). Twelve 900-μl fractions were collected for each experiment. From 500 μl of each fraction, proteins were precipitated with trichloroacetic acid. Fifty nanograms of LysA RNA (Bacillus subtilis) [70] was added to 100 μl of each fraction, and total RNA was isolated with RNeasy Mini columns (Qiagen) including on-column DNase I digestion. For microarray studies, 500 μl of fractions containing 60S subunits, the 80S monosome, and polysomes were pooled. RNA was precipitated with 2.25 M guanidinium-HCl (final concentration)/50% ethanol overnight at −20 °C and purified as described above. Total RNA was finally precipitated with 1.5 M LiCl and washed with ethanol to remove residual heparin.

### Affinity purification of ribosomes.

A detailed protocol is available in Text S2 and at our Web site (http://gerber.openwetware.org/). In brief, rabbit IgGs (Sigma) were coupled to carboxylated polybeads (0.75–1 μm; Polysciences) by shaking at 850 rpm overnight. The beads were washed three times for 30 min with blocking buffer (Polysciences) supplemented with 0.4 mg/ml heparin and 0.1 mg/ml Escherichia coli tRNA (Sigma). Two-milliliter blocked 2.5% (w/v) polybead solution was used for each affinity purification. Beads were collected by centrifugation (5 min, 6 K rpm, RT), and incubated with 750 μl of cell-free extract (∼7.5 mg/ml protein) for 2 h at 4 °C by shaking at 850 rpm. The beads were then collected by centrifugation (2 min, 6 K rpm, RT) and washed four times for 15 min in buffer C (20 mM Tris-HCl [pH 8.0], 140 mM KCl, 2 mM MgCl_2_, 5% glycerol, 0.5 mM DTT, 40 U/ml RNase Out [Invitrogen]). Ribosomes were released from beads by incubation with 750 μl of buffer C supplemented with 0.8 U/μl TEV protease (Invitrogen) for 2 h at 15 °C. Beads were pelleted by centrifugation, and the supernatant containing purified ribosomes was collected and frozen in liquid nitrogen. RNA was isolated from cell-free extracts (total RNA) and from affinity-purified ribosomes with RNeasy Micro columns including on-column DNase I digestion (Qiagen).

### Mass spectrometric analysis.

Affinity-purified ribosomes were separated on Tris-glycine gels, stained with Coomassie Blue G-250 (BioRad), and 11 regions covering regions from 10–75 kDa of the gel were excised. Gel bands were cut into pieces, which were subsequently washed twice with 100 μl of 100 mM NH_4_HCO_3_/50% acetonitrile and once with 50 μl of acetonitrile. Gel bands were suspended in 20 μl of buffer D (10 mM Tris-HCl [pH 8.2], 2 mM CaCl_2_) containing 5 ng/μl trypsin (Roche Applied Science; recombinant, proteomics grade) and digested overnight at 37 °C. Supernatants were collected, and gel pieces were repeatedly extracted with 100 μl of 0.1% trifluoroacetic acid/50% acetonitrile. The supernatants were combined, dried, and peptides redissolved in 25 μl of 0.1% formic acid. Three microliters of this peptide solution were analyzed by liquid chromatography–tandem mass spectrometry (LC-MS/MS) (QTOF Ultima API connected online to a nanoAcquity UPLC [Waters]). For shotgun analysis, 80 μl of affinity-purified ribosomes (10–15 μg) were precipitated with trichloroacetic acid, and pellets were dissolved in 50 μl of buffer D (10 mM Tris-HCl [pH 8.2], 2 mM CaCl_2_) containing 0.5 μg trypsin. After digestion at 37 °C overnight, samples were dried and redissolved in 50 μl of 0.1% formic acid, and 0.5 μl were analyzed by LC-MS/MS. Database searches were performed by using the in-house Mascot search engine (UniRef100 database without taxonomy filter) (http://www.matrixscience.com). Scaffold (Proteome Software) was used to validate protein identifications derived from MS/MS sequencing results. Scaffold verifies peptide identifications assigned by SEQUEST and Mascot using the X!Tandem database searching program [71]. Scaffold then probabilistically validates these peptide identifications using PeptideProphet [72] and derives corresponding protein probabilities [73].

### DNA microarrays.

Oligonucleotide arrays were used for the comparative analysis of mRNAs associated with Rpl16a, Rpl16b, and of polysomal fractions (Dataset S1). These arrays contain 70-mer oligo probes for 10,944 features combined from the Array-Ready Oligo Set Version 1.1 representing 6,388 S. cerevisiae ORFs, and the Yeast Brown Lab Oligo Extension Version (YBOX vers. 1.0) with 3,456 probes for all noncoding RNAs, introns, mitochondrial genes, and diverse controls (Operon). The probes were printed on epoxy-coated glass slides (Nexterion slide E) at the Center for Integrative Genomics, University of Lausanne, Switzerland. Oligo arrays were blocked in 5× SSC, 0.1 mg/ml BSA, 0.1 % SDS for 1 h at 42 °C, and subsequently washed three times in 0.1× SSC for 5 min at room temperature, rinsed in water for 30 s, and dried by centrifugation (500×*g* for 2 min). The slides were used the same day.

DNA microarrays containing PCR-derived probes for all S. cerevisiae ORFs, introns, and the mitochondrial genome were used for the analysis of changes in the global transcript levels and ribosome associations upon diverse stress treatments. These arrays consisted of 7,892 features representing 6,954 ORFs and were processed as previously described [74]; http://cmgm.stanford.edu/pbrown/protocols/index.html).

### Microarray hybridization and data analysis.

Equal amounts of a pool of five synthetically prepared B. subtilis RNAs [70] (DNA microarrays) or 1 μl of a pool of eight control RNAs (2.5–50 pg, Ambion ArrayControls; for oligo arrays) were added to each RNA sample prior to labeling as a control for the labeling procedure.

To analyze the enrichment of transcript with Rpl16a or Rpl16b, 10 μg of total and Rpl16a/b-associated RNAs were labeled with Cy3 and Cy5 fluorescent dyes, respectively, following cDNA synthesis with amino-allyl dUTP in addition to the four natural dNTPs and oligo(dT_20_V) primers. Likewise, 10 μg of total and of RNAs isolated from the 60S, 80S, and polysomal fractions were labeled with Cy3 and Cy5 fluorescent dyes, respectively, to analyze enrichment of messages in the corresponding fractions of the sucrose density gradient. The samples were mixed and competitively hybridized on oligo arrays for 15 h at 42 °C in MWG formamide buffer (Ocimum Biosolutions) supplemented with 10 μg of polyadenosines (Sigma). The arrays were washed in 400 ml of three subsequent wash buffers made of 2× SSC, 0.2% SDS (bath 1), 2× SSC (bath 2), and 0.02× SSC (bath 3). The first wash was performed at 42 °C for 13 min (1 min by manual shaking followed by 12 min on a shaker), and the following washes at room temperature for 12 min each. Slides were briefly immersed in 100% ethanol, and dried by centrifugation.

To monitor changes in the global transcript levels and ribosome associations under diverse stress conditions, fluorescently labeled cDNA samples were prepared from 5 μg of RNA as described above. Cy5-labeled samples prepared from total RNA or Rpl16a-associated mRNA were competitively hybridized to Cy3-labeled cDNA derived from a common reference RNA on DNA microarrays for 15 h at 65 °C. The reference RNA is a mixture of equal amounts of total RNA isolated from yeast grown to mid-log phase in five different media: YPD, YP with 2% galactose, YP with 2% glycerol, SC dextrose, and SC with 2% galactose.

All microarrays were scanned with an Axon Instruments Scanner 4200A (Molecular Devices). Scanning parameters were adjusted to give similar fluorescent intensities control spots in both channels. Data were collected with the GENEPIX 5.1 (Molecular Devices) and spots with abnormal morphology were excluded from further analysis. Arrays were computer normalized by the Stanford Microarray Database (SMD) [75]. Log_2_ median ratios were retrieved from SMD, and data were exported into Microsoft Excel after filtering for regression correlation of greater than 0.5 (filters for large variations in the ratios of pixels within each spot), and signal over background greater than 1.6. Array data are deposited at SMD and at the Gene Expression Omnibus (GEO) with accession number GSE13682.

Data from two independent biological Rpl16a and Rpl16b affinity purifications (total of four arrays), and from four independent polysomal fractionations analyzing RNAs in 60S, 80S, and polysomal fractions were collected, and data for 7,316 features were further analyzed with Excel (Dataset S1). The Pearson correlations between the experiments were determined on normalized log_2_ median ratios with Excel.

To generate a dataset representing changes of transcript levels and ribosome-associations of stress-treated cells, we performed five biological replicates of untreated control cells (time zero, *t* = 0), three replicates of cells after amino acid starvation and osmotic shock, and four replicates following CFW and menadione treatments (total of 52 arrays, Dataset S2). The median log_2_ ratios for genes with data from at least three independent replicates were averaged and normalized to the average of untreated control samples. In total, we obtained data for 7,062 arrayed features representing 6,350 genes. To generate a list with significantly changed genes, we selected all features that were on average at least 2-fold changed (Δlog_2_ ratio ≥ 1) with *p*-value ≤ 0.05 (Student *t*-test). Normalized data for 1,458 significantly changed nuclear-encoded and mitochondrial messages are listed in Dataset S3. A hierarchical cluster of these genes and arrays was generated with Cluster 3.0 [46] and visualized with Java TreeView 1.0.12 [76]. Commonly enriched GO terms among list of genes were retrieved with GO Term Finder that uses a hypergeometric distribution with Multiple Hypothesis Correction (i.e., Bonferroni correction) to calculate *p*-values (*Saccharomyces* Genome Database [SGD]; http://www.yeastgenome.org).

### SYBR Green real-time PCR analysis.

Reverse transcription was performed with the High-Capacity cDNA Reverse Transcription Kit (Applied Biosystems) and 25 ng of total RNA from yeast cell extracts or 5–500 ng of total RNA isolated from fractions of the sucrose density gradient. Quantitative PCR was performed with the SYBR Green PCR Master Mix (Applied Biosystems) in an Applied Biosystems 7900HT Fast Real-Time PCR System. The following temperature profile was applied: 50 °C for 2 min; 95 °C for 15 min; 40 cycles of the sequence 95 °C for 15 s; 58 °C for 1 min; 95 °C for 15 s; 60 °C for 15 s; 95 °C for 15 s. Ct-values were either normalized to the actin (ACT1) control probe or to the LysA control RNA (LYSA) that was added to each fraction from polysomal gradients.

### Purification of mitochondria and ATPase assays.

Cells were grown in SC media to mid-log phase and treated either with 10 μg/ml CFW or solvent only (water:ethanol = 4:1) for 20 min. To inhibit translation elongation during drug exposure, 0.1 mg/ml CHX was added to cultured cells 10 min prior to drug addition, and 0.1 mg/ml CHX was added concomitantly with the indicated drugs or controls to the cells. Cells were collected by centrifugation (3.5 K rpm, 5 min, RT) and washed twice with water containing 0.1 mg/ml CHX. Mitochondria were isolated as previously described with minor modifications [77]. In brief, cells were resuspended in 1 ml of spheroblast buffer (1.2 M sorbitol, 20 mM HEPES [pH 8], 0.1 mg/ml CHX, 10 mM DTT) containing 200 U lyticase (Sigma) and incubated for 22 min at 30 °C. After centrifugation (5,000*g*, 5 min, RT), the cells were dissolved in 500 μl of ice-cold mitochondrial isolation buffer (MIB; 0.6 M sorbitol, 10 mM HEPES [pH 8], 0.1 mg/ml CHX, 0.5 mM PMSF) and broken mechanically with one-third volume of 0.5-mm glass beads by vortexing for 40 s. Cell lysates were centrifuged for 5 min at 5,000*g* at 4 °C, beads were washed with another 500 μl of MIB, and supernatants were pooled. After clearing the lysate (5,000*g*, 5 min, 4 °C), 900 μl of the extract were centrifuged (13.2 K rpm, 10 min, 4 °C), and the pelleted mitochondria were washed twice with 500 μl of MIB. Purified mitochondria were resuspended in 50 μl of MIB and 10 μl of 50% glycerol.

ATPase activity was measured with a colorimetric assay according to the manufacturer's instructions (ATPase Assay Kit; Innova Biosciences). Purified mitochondria were tested for contamination by free inorganic phosphate (P_i_). Ten OD_280_ units of mitochondria were preincubated with 2 μg of F_1_-ATPase inhibitor oligomycin (Sigma; referred to as the oligomycin control) or methanol in 100 μl of bidistilled water for 10 min at room temperature. ATP hydrolysis was started by addition of 100 μl of Substrate-buffer mix (SB) supplemented with 0.5 mM ATP. After 15 min, the reaction was stopped with 50 μl of Gold mix, and the absorbance at 635 nm was measured after an additional 30 min. Measuring the changes in absorbance allows the quantitative determination of released P_i_ during the ATPase reaction. To calculate mitochondrial ATPase activity, the average changes in absorbance of oligomycin control reactions were subtracted from changes in absorbance from reactions without oligomycin (all in duplicate). The activities of drug-treated cells were compared to solvent-only–treated cells, and *p*-values were calculated with the Student *t*-tests (two-tailed, two-sample).

### Accession numbers.

Array data are deposited at SMD and at the Gene Expression Omnibus (GEO) with accession number GSE13682. The accession numbers for S. cerevisiae genes are from SGD (http://www.yeastgenome.org/) (ORF/SGD identification number): *ACT1* (YFL039C/ S000001855), *ASC1* (YMR116C/S000004722), *ATP4* (YPL078C/S000005999), *ECM33* (YBR078W/S000000282), *GCN4* (YEL009C/S000000735), *HXK2* (YGL253W/S000003222), *INO80* (YGL150C/S000003118), *MAL31* (YBR298C/S000000502), *MDH1* (YKL085W/S000001568), *RPL16A* (YIL133C/S000001395), *RPL16B* (YNL069C/S000005013), *RPL25* (YOL127W/S000005487), *RRD1* (YIL153W/S000001415), *RRN7* (YJL025W/S000003562), *SUT1* (YGL162W/S000003130), *TIM11* (YDR322C-A/S000007255), *UTH1* (YKR042W/S000001750), *YHR113* (YHR113/S000001155), and *YIM1* (YMR152W/S000004760).

## Supporting Information

Dataset S1 Profiles of Rpl16a- and Rpl16b-Bound RNAs and of Polysomal RNAs Cy5/Cy3 fluorescence ratios from microarray hybridizations analyzing Rpl16a/b-associated RNAs or RNAs present in corresponding sucrose gradient fractions versus total RNA (for a histogram of the data, see Figure S2). Columns indicate the following (from left to right): YORF; ID, array identification number; type of oligo; gene name; GO annotations; log_2_ ratios of independent Rpl16a affinity isolations; average log_2_ ratio of Rpl16a affinity isolations; average percentile ranks; log_2_ ratios of independent Rpl16b affinity isolations; average log_2_ ratio of Rpl16b affinity isolations; average percentile ranks; log_2_ ratios of four independent sucrose gradient isolations; average log_2_ ratio of sucrose gradient isolations; and average percentile ranks. A key to the type of oligos, and the Pearson correlation of profiles are given in separate worksheets.
(2.03 MB XLS)

Dataset S2 Microarray Data of Different Stress Treatments Each worksheet contains normalized data sampling relative changes of total RNA expression (Total) or ribosome associations (RA) of stress-treated cells and of untreated cells (*t* = 0; see Materials and Methods for experimental design). Worksheets are designated the following: AA10: amino acid starvation for 10 min; AA20: amino acid starvation for 20 min; Sorbitol; CFW100: treatment with 100 μg/ml CFW; CFW10: treatment with 10 μg/ml CFW; Menadione. Columns in each worksheet indicate the following (from left to right): YORF; gene name; GO annotations; log_2_ ratio (expression of sample versus common reference) of three to four independent biological replicates; average log_2_-ratio from at least three independent experiments; log_2_ ratios (sample versus reference) of five biological replicates from untreated (*t* = 0) cells; average log_2_ ratio of at least three *t* = 0 experiments; average log_2_ ratios of untreated control samples subtracted from average log_2_ ratios of stress-treated samples; *p-*values comparing stress-treated versus untreated controls determined with a two-tailed, unpaired Student *t*-test.
(16.87 MB XLS)

Dataset S3 List of Genes That Significantly Changed upon Stress Raw data of the cluster shown in Figure 2B including gene annotations (YORF; gene name; and GO annotation).
(326 KB XLS)

Figure S1 Rpl16a- and Rpl16b-Deficient Cells Show No Different Growth Sensitivities Evaluation of growth sensitivity to CFW of *rpl16aδ* and *rpl16bδ* cells 3 d after plating a series of 10-fold diluted spots in synthetic complete medium (SC).
(4.10 MB TIF)

Figure S2 Enrichment Profiles of Rpl16a/b-ZZ–Associated mRNAs (A) Scatter plot of average Cy5/ Cy3 fluorescence ratios (log_2_) from microarray hybridizations comparing Rpl16a-ZZ (*x*-axis), and Rpl16b-ZZ (*y*-axis). The ratio of the two RNA populations at a given array element reflects the enrichment of the respective mRNA by ribosome purification. Marked in blue are 62 features (30 genes) preferentially associated with Rpl16a-ZZ (Δlog_2_ ≥ 1); in yellow 25 features (17 genes) with Rpl16b-ZZ (Δlog_2_ ≤ −1). R: correlation coefficient. HAC1 mRNA is depicted by an arrow.
(B) Distribution of average Cy5/ Cy3 fluorescence ratios from two independent microarrays hybridizations analyzing Rpl16a- and Rpl16b-associated RNAs (Dataset S1). The frequency distribution of all analyzed features is shown in black/grey and refers to the left *y*-axis. The distribution of Ty elements is colored in green, the distribution of introns in red, and both refer to the right *y*-axis.
(6.18 MB TIF)

Figure S3 Polysomal Profiles of Stress-Treated Cells Polysomal profiles of untreated cells grown in SC medium (*t* = 0), and of cells subjected to specific stress treatments. The A_254_ ratio of 60S subunits to polysomes represents a rough estimate for global translational activity.
(1.28 MB TIF)

Figure S4 Stress-Dependent Correlations of Global Transcript Levels and Translatome Relative changes of mRNA expression (total RNA) compared with their ribosome associations (RA) in stress-treated versus untreated cells. Average Cy5/Cy3 fluorescence ratios from microarray hybridizations comparing steady-state mRNA levels from treated versus untreated cells are plotted on the *x*-axis, and the respective changes of ribosome-associations are plotted on the *y*-axis. The blue points refer to the group of messages with preferentially altered mRNA levels (group T, Figure 2); the yellow points refer to mRNAs preferentially changed at translatome (group R, Figure 2); and green points represent messages that are significantly changed at both global transcript levels and translatome (group H, Figure 2). The grey points refer to messages that are not considered to be significantly altered by our arbitrary cut-off (>2-fold, *p* < 0.05). R: correlation coefficient.
(1.18 MB TIF)

Figure S5 Functional Themes among Differentially Regulated Messages in Response to Stress Changes in ribosome associations in response to stress treatment were normalized to changes of total mRNA steady-state levels (ΔΔlog_2_ ratios). Genes that differed more than 2-fold (number of genes indicated in brackets next to the applied stress) were searched for common GO terms. The significance of enrichment of the GO term is represented as a heat map in which the color code corresponds to *p*-values (see Figure 3).
(1.07 MB TIF)

Figure S6 Polysomal Distribution of SUT1 mRNA in Response to 1 M Sorbitol See Figure 4 for a description. SUT1 mRNA is shown in blue; ACT1 mRNA is the negative control shown in grey.
(1.37 MB TIF)

Figure S7 Immunoblot Analysis Following the Purification of Mitochondria Samples were probed with specific antibodies detecting Zwf1p, a cytoplasmic protein (57.5 kDa), and Por1p (30.4 kDa), a mitochondrial membrane protein. An asterisk (*) indicates a second band detected with anti-Zwf1 antibodies that is of unknown origin. Lanes: Ex, extract; P, pelleted mitochondria; Sup, supernatant after high-speed centrifugation to pellet mitochondria.
(1.73 MB TIF)

Table S1 Mass Spectrometric Analysis of Affinity-Purified Ribosomes Proteins were analyzed by LC-MS/MS either after separation by SDS-PAGE (gel) or directly after purification and TCA-precipitation (Shot Gun). Both ORFs are indicated for paralogous ribosomal proteins. The likelihood (in percent) for correct identification of the protein was calculated with Scaffold.
(21 KB XLS)

Text S1 Functional Themes among Stress-Regulated Messages (34 KB DOC)

Text S2 Protocol for Affinity Purification of Ribosomes (46 KB DOC)

## Abbreviations

CFW: Calcofluor-white
CHX: cycloheximide
ESR: environmental stress response
GO: Gene Ontology
IgG: immunoglobulin G
LC-MS/MS: liquid chromatography–tandem mass spectrometry
MS: mass spectrometry
qPCR: quantitative polymerase chain reaction
RBP: RNA-binding protein
SMD: Stanford Microarray Database
TEV: tobacco-etch virus
