# Correction: Stress-Dependent Coordination of Transcriptome and Translatome in Yeast

**DOI:** 10.1371/annotation/7462bca2-5358-43c8-be2e-94e8a8f46159

**Published:** 2012-01-09

**Authors:** Regula E Halbeisen, André P Gerber

The column headings in Dataset S3 are incorrect due to a formatting error. The Column headings in Columns K-P were originally presented in an incorrect order. Please view the correct Dataset S3 here: 
