# Supplementary figures and images for: Stress-Dependent Coordination of Transcriptome and Translatome in Yeast
Source: PLoS Biol. 2009 May 5;7(5):e1000105. doi: 10.1371/journal.pbio.1000105 (PMC2675909; doi:10.1371/journal.pbio.1000105)

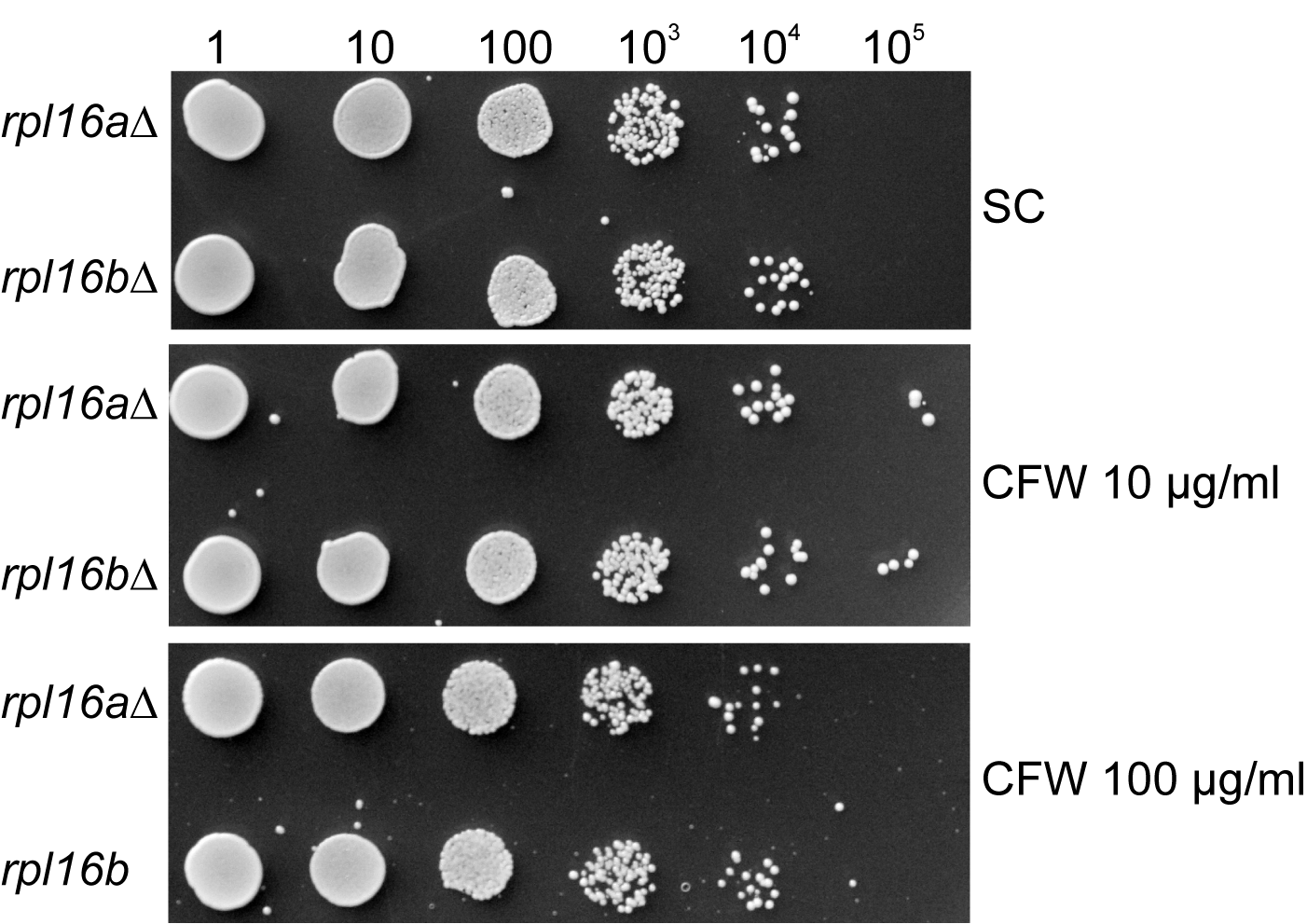

Supplement: Figure S1 — Evaluation of growth sensitivity to CFW of rpl16aδ and rpl16bδ cells 3 d after plating a series of 10-fold diluted spots in synthetic complete medium (SC). (4.10 MB TIF) [file pbio.1000105.sg001.tif]

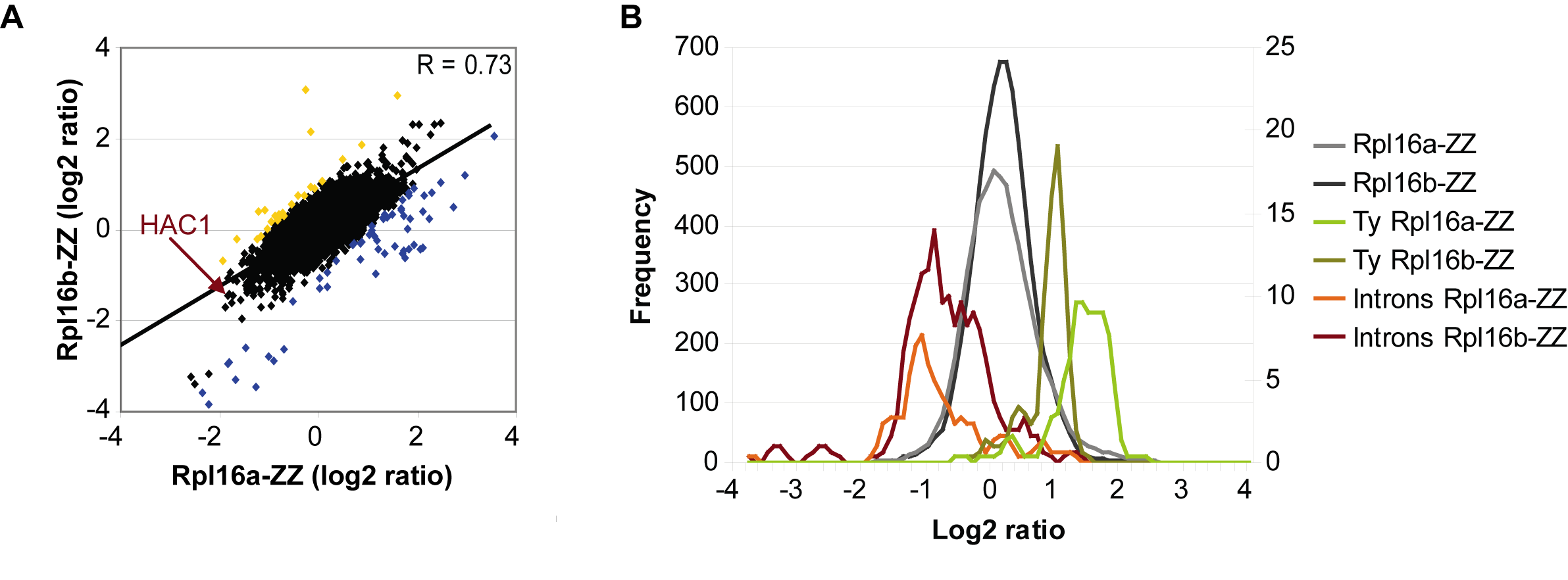

Supplement: Figure S2 — (A) Scatter plot of average Cy5/ Cy3 fluorescence ratios (log2) from microarray hybridizations comparing Rpl16a-ZZ (x-axis), and Rpl16b-ZZ (y-axis). The ratio of the two RNA populations at a given array element reflects the enrichment of the respective mRNA by ribosome purification. Marked in blue are 62 features (30 genes) preferentially associated with Rpl16a-ZZ (Δlog2 ≥ 1); in yellow 25 features (17 genes) with Rpl16b-ZZ (Δlog2 ≤ −1). R: correlation coefficient. HAC1 mRNA is depicted by an arrow. (B) Distribution of average Cy5/ Cy3 fluorescence ratios from two independent microarrays hybridizations analyzing Rpl16a- and Rpl16b-associated RNAs (Dataset S1). The frequency distribution of all analyzed features is shown in black/grey and refers to the left y-axis. The distribution of Ty elements is colored in green, the distribution of introns in red, and both refer to the right y-axis. (6.18 MB TIF) [file pbio.1000105.sg002.tif]

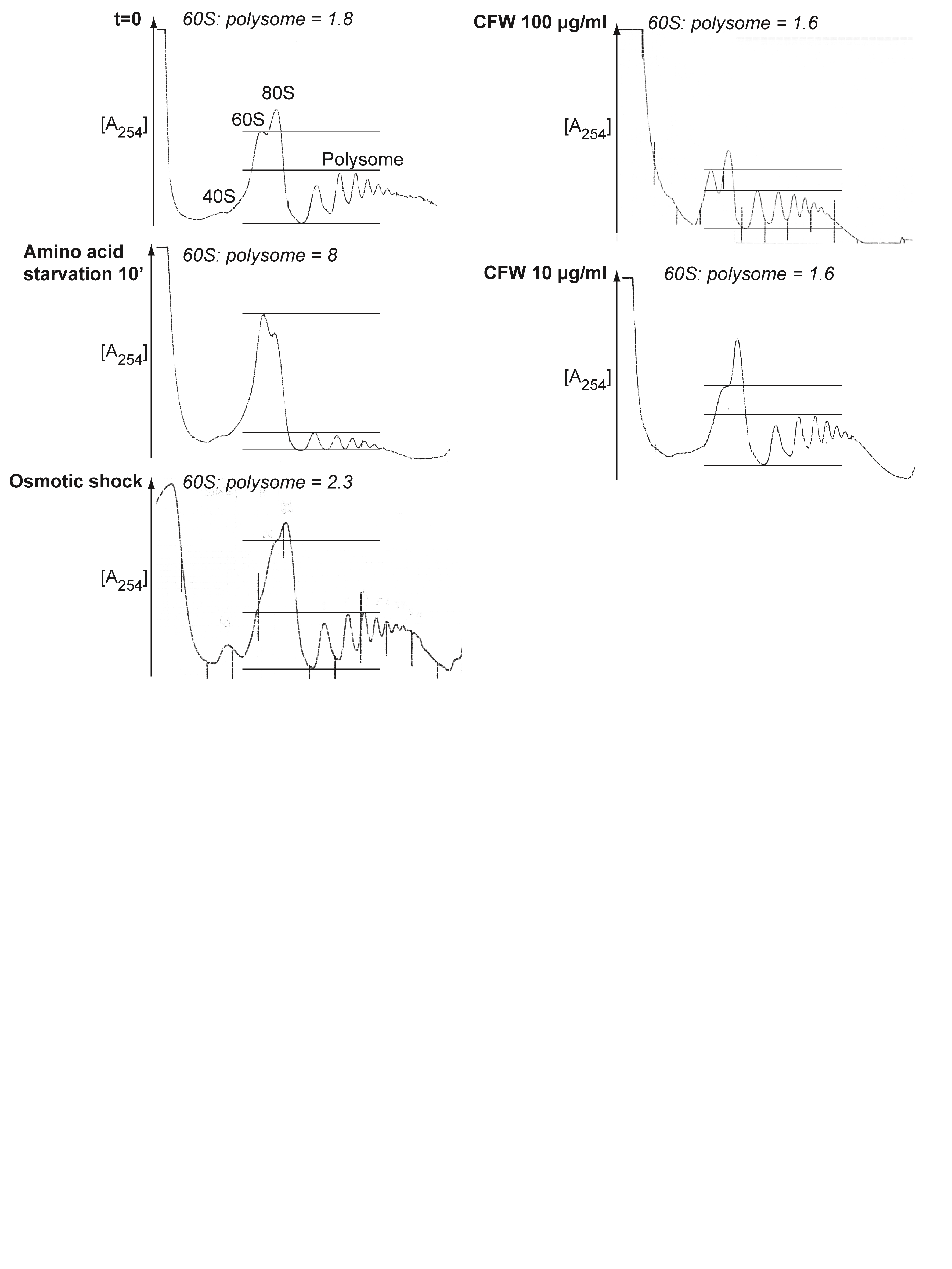

Supplement: Figure S3 — Polysomal profiles of untreated cells grown in SC medium (t = 0), and of cells subjected to specific stress treatments. The A254 ratio of 60S subunits to polysomes represents a rough estimate for global translational activity. (1.28 MB TIF) [file pbio.1000105.sg003.tif]

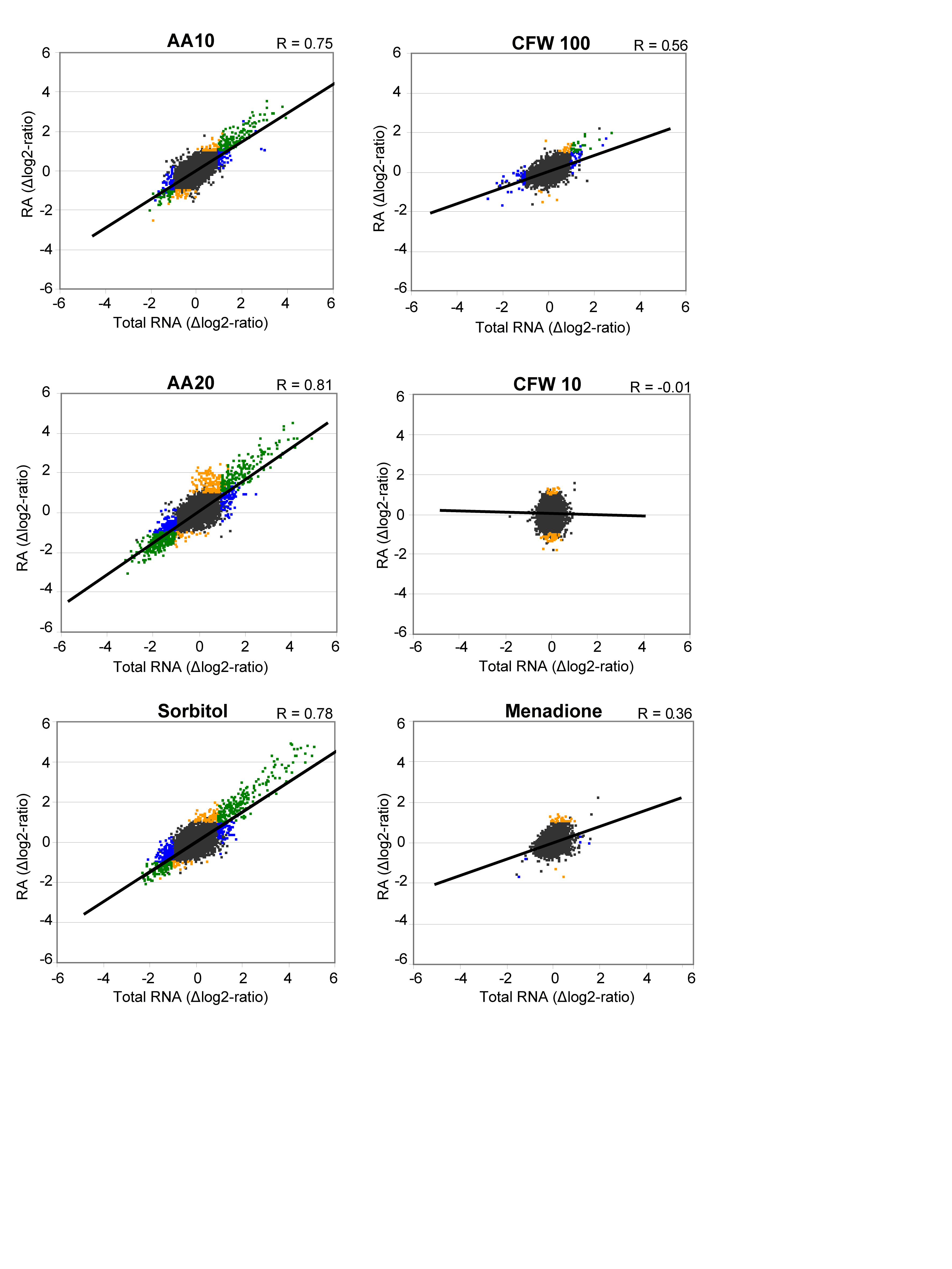

Supplement: Figure S4 — Relative changes of mRNA expression (total RNA) compared with their ribosome associations (RA) in stress-treated versus untreated cells. Average Cy5/Cy3 fluorescence ratios from microarray hybridizations comparing steady-state mRNA levels from treated versus untreated cells are plotted on the x-axis, and the respective changes of ribosome-associations are plotted on the y-axis. The blue points refer to the group of messages with preferentially altered mRNA levels (group T, Figure 2); the yellow points refer to mRNAs preferentially changed at translatome (group R, Figure 2); and green points represent messages that are significantly changed at both global transcript levels and translatome (group H, Figure 2). The grey points refer to messages that are not considered to be significantly altered by our arbitrary cut-off (>2-fold, p < 0.05). R: correlation coefficient. (1.18 MB TIF) [file pbio.1000105.sg004.tif]

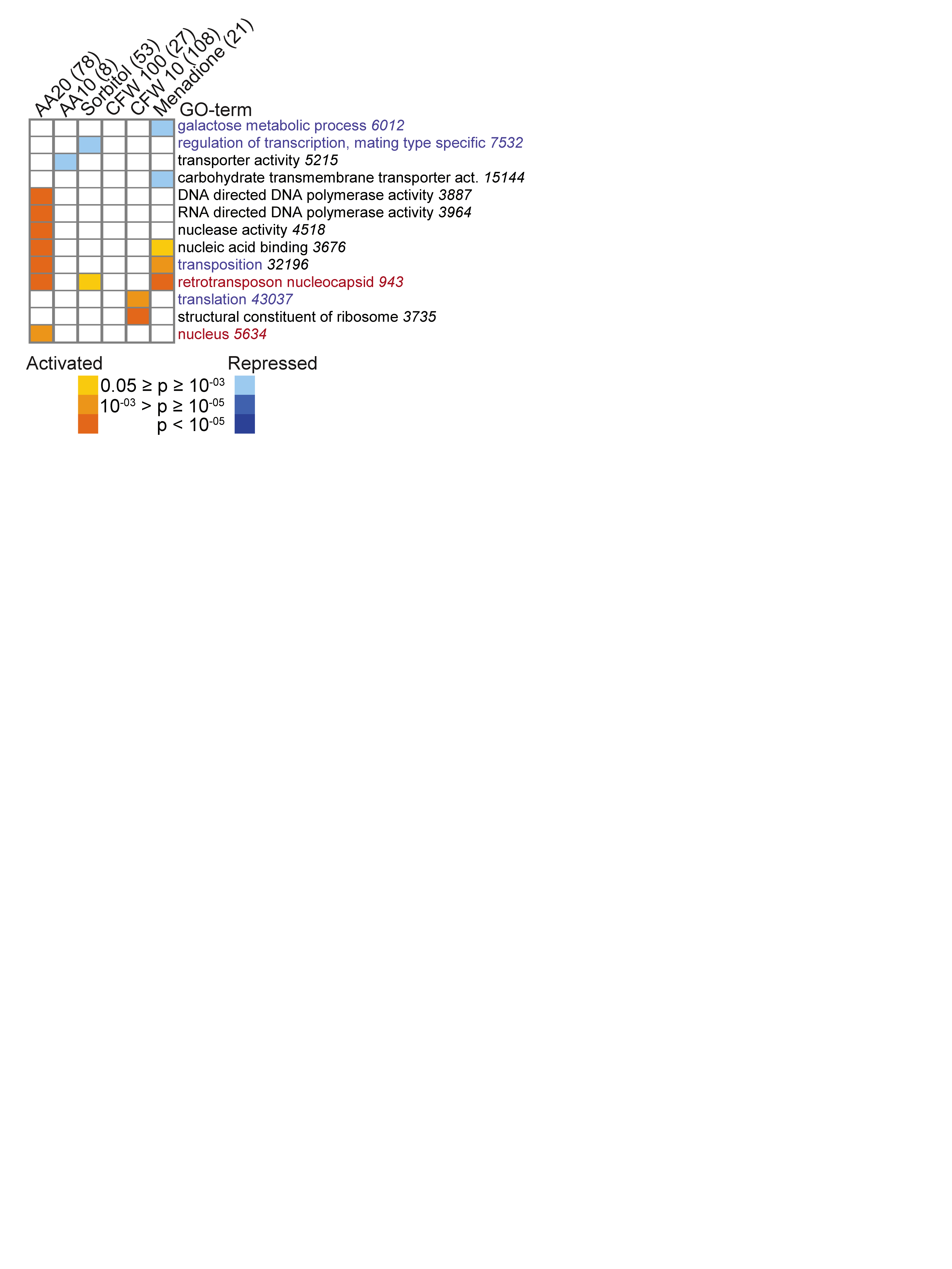

Supplement: Figure S5 — Changes in ribosome associations in response to stress treatment were normalized to changes of total mRNA steady-state levels (ΔΔlog2 ratios). Genes that differed more than 2-fold (number of genes indicated in brackets next to the applied stress) were searched for common GO terms. The significance of enrichment of the GO term is represented as a heat map in which the color code corresponds to p-values (see Figure 3). (1.07 MB TIF) [file pbio.1000105.sg005.tif]

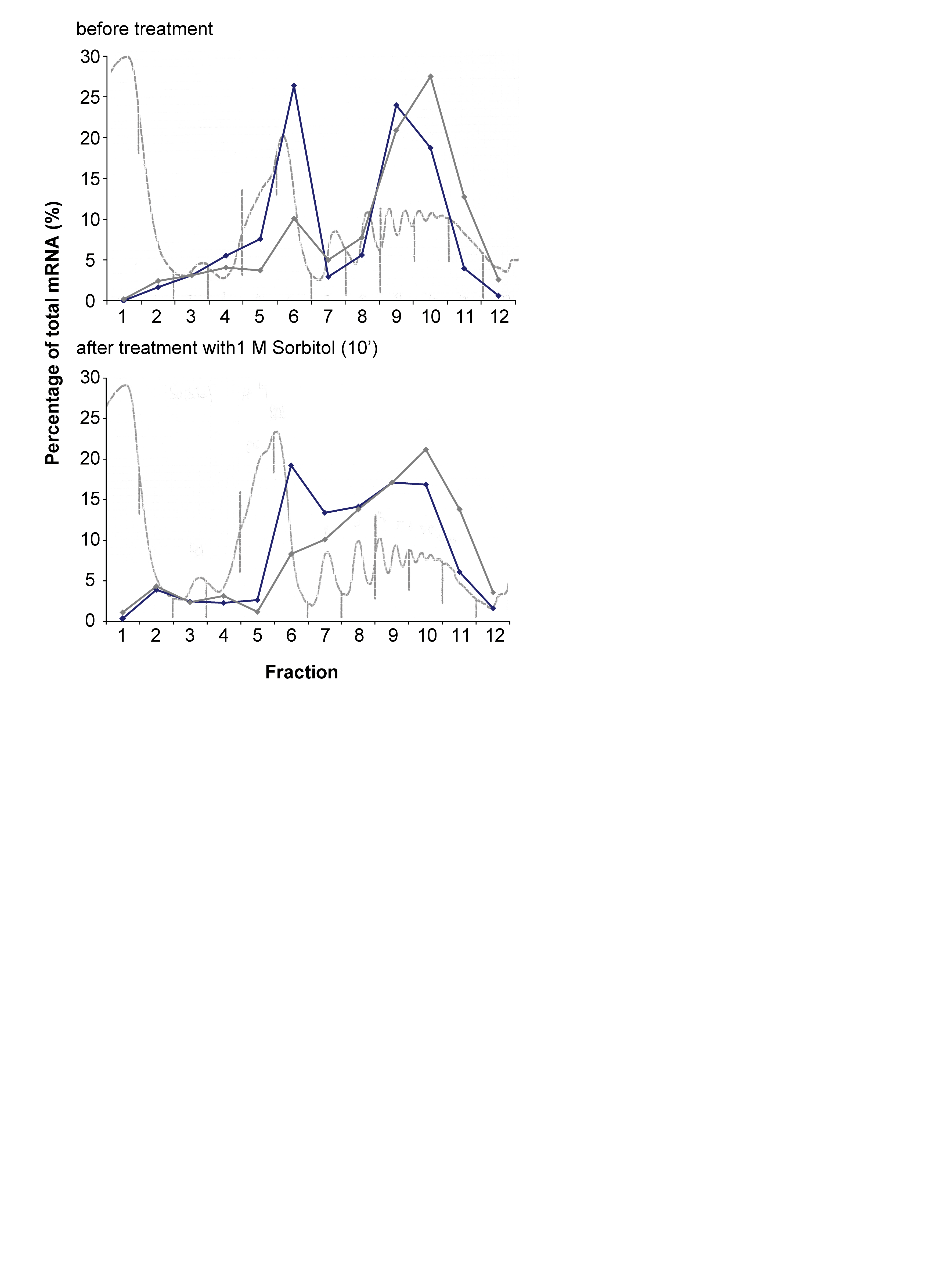

Supplement: Figure S6 — See Figure 4 for a description. SUT1 mRNA is shown in blue; ACT1 mRNA is the negative control shown in grey. (1.37 MB TIF) [file pbio.1000105.sg006.tif]

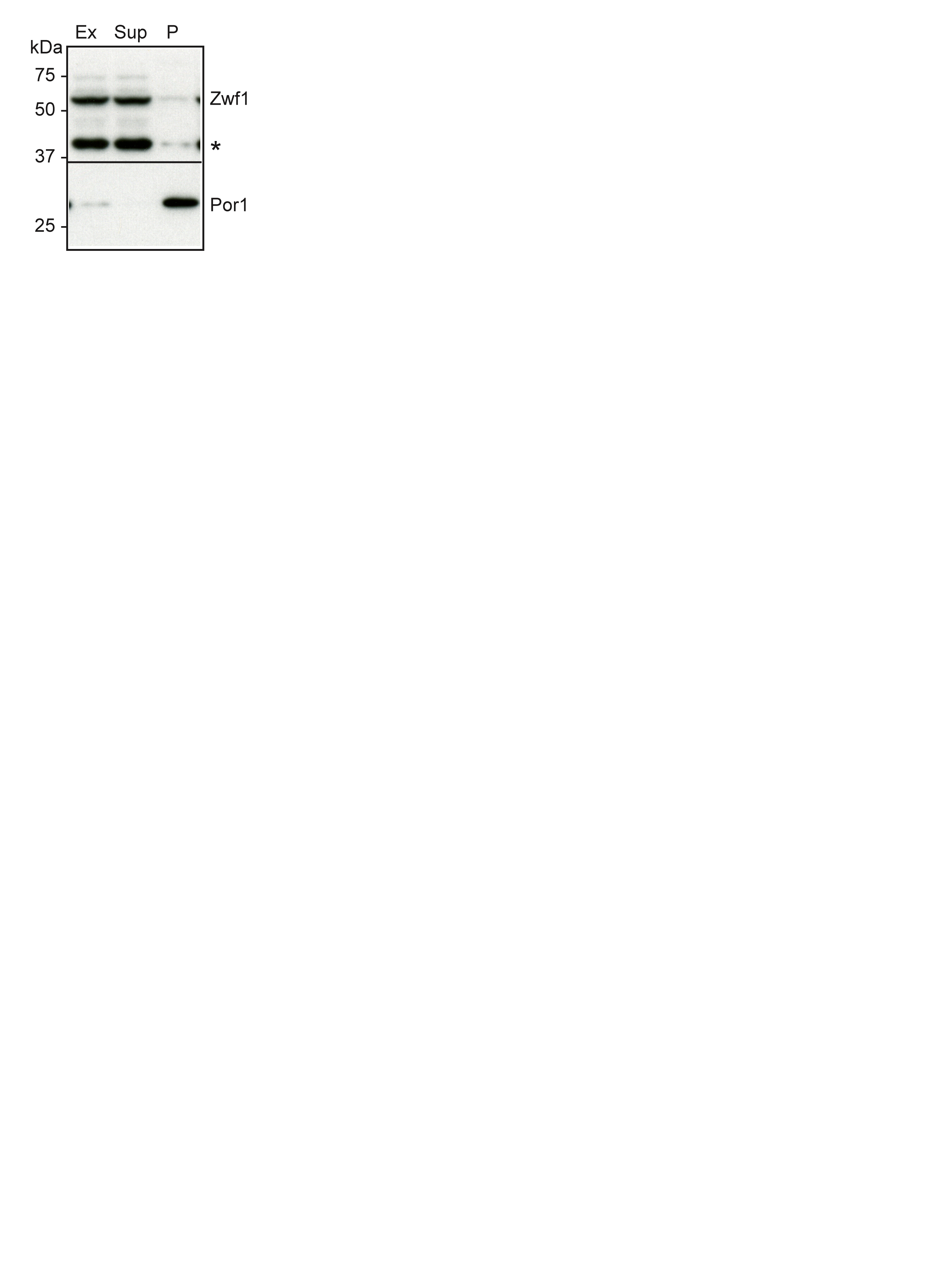

Supplement: Figure S7 — Samples were probed with specific antibodies detecting Zwf1p, a cytoplasmic protein (57.5 kDa), and Por1p (30.4 kDa), a mitochondrial membrane protein. An asterisk (*) indicates a second band detected with anti-Zwf1 antibodies that is of unknown origin. Lanes: Ex, extract; P, pelleted mitochondria; Sup, supernatant after high-speed centrifugation to pellet mitochondria. (1.73 MB TIF) [file pbio.1000105.sg007.tif]
