# Supplementary material for: Stress-Dependent Coordination of Transcriptome and Translatome in Yeast
Source: PLoS Biol. 2009 May 5;7(5):e1000105. doi: 10.1371/journal.pbio.1000105 (PMC2675909; doi:10.1371/journal.pbio.1000105)
Supplement: Text S1 — (34 KB DOC) [file pbio.1000105.sd004.doc]

**Text S1. Functional themes among stress-regulated messages.**

The genes present in the six subclusters (I-VI) of Figure 2B were searched for commonly enriched GO terms. Subcluster I contains Ty-elements, a transposable element in yeast (p < 10^-37^). Amino acid starvation for 20 minutes but not for 10 minutes, as well as menadione treatment increased the ribosome association of Ty-elements, but respective total mRNA levels remained unchanged. It is known that environmental changes such as low temperature, UV irradiation or starvation induces transposition [1-3]. Nevertheless, it was unexpected to find Ty-elements to become preferentially induced at the translatome indicating the potential for translational regulation of those elements. Not surprisingly amino acid starvation increased the expression of genes involved in amino acid metabolic process (p < 10^-33^) or organic acid transport (p < 10^-5^) to substitute for starvation (subcluster II), while osmotic shock selectively induced genes coding for proteins that play a role in stress response (p < 10^-4^) and hexose transport (p < 0.02), which may be required to counterbalance the osmotic pressure (subcluster III). Both, amino acid starvation and osmotic shock, also induced a set of common genes coding for proteins involved in organic acid metabolic process (p < 2 x 10^-4^) or oxidoreductase activity (p < 7 x 10^-4^, subcluster IV). Under very mild stress conditions (CFW 10 µg/ml, menadione), these genes are weakly upregulated in the translatome (average log_2_ ratio = 0.35/ 0.25) but not in the transcriptome profiles (average log_2_ ratio = 0.03/ 0.13). Possibly translation is initially activated through the efficient recruitment of cytoplasmic mRNAs to the ribosomes. Interestingly, a fraction of these genes (33 of 133) are part of environmental stress response (ESR) genes [4]. All severe stresses strongly downregulated messages coding for proteins involved in translation and ribosome biogenesis/ assembly (p < 10^-105^) (subclusters V and VI). The repression of ribosomal protein genes occurs under diverse stress conditions and is accompanied by immediate stop of cell growth [4,5]. 20 minutes of amino acid starvation had a more pronounced effect on selected genes involved in translation compared to the 10-minute treatment (subcluster V). 92% and 87% of all genes that were selectively changed in transcriptome and translatome after 10 minutes (subcluster V) exhibited an increased amplitude after 20 minutes. In contrast, under all conditions of mild stress, the genes present in the subclusters IV and V were not repressed. Upon CFW (10 µg/ml) treatment, the genes present in these subclusters even exposed a higher ribosome association. This is corroborated by a slight increase in polysomes as seen in polysomal profiles (Figure S2, 60S: polysome = 1.6). Notably, cells treated with 100 µg/ml CFW showed an intermediate behavior between mildly and severely stressed cells with only marginally correlated profiles compared to the low dose of the drug (Pearson correlations p = 0.17 at the transcriptome, p = 0.36 at the translatome), indicating that different, and dose-dependent programs were activated.

1. Bradshaw VA, McEntee K (1989) DNA damage activates transcription and transposition of yeast Ty retrotransposons. Mol Gen Genet 218: 465-474.

2. Paquin CE, Williamson VM (1984) Temperature Effects on the Rate of Ty Transposition. Science 226: 53-55.

3. Ribeiro-dos-Santos G, Schenberg AC, Gardner DC, Oliver SG (1997) Enhancement of Ty transposition at the ADH4 and ADH2 loci in meiotic yeast cells. Mol Gen Genet 254: 555-561.

4. Gasch AP, Spellman PT, Kao CM, Carmel-Harel O, Eisen MB, et al. (2000) Genomic expression programs in the response of yeast cells to environmental changes. Mol Biol Cell 11: 4241-4257.

5. Warner JR (1999) The economics of ribosome biosynthesis in yeast. Trends Biochem Sci 24: 437-440.
