# Supplementary material for: Stress-Dependent Coordination of Transcriptome and Translatome in Yeast
Source: PLoS Biol. 2009 May 5;7(5):e1000105. doi: 10.1371/journal.pbio.1000105 (PMC2675909; doi:10.1371/journal.pbio.1000105)
Supplement: Text S2 — (46 KB DOC) [file pbio.1000105.sd005.doc]

**Text S2. Protocol for affinity-purification of ribosomes**

**Coupling of polybeads with the carbodiimide kit for carboxylated microparticles (Polysciences)**

**Contents of kit:**

Buffer 1 0.1 M carbonate buffer

Buffer 2 0.02 M phosphate buffer, pH 4.2

Bottle 3 carbodiimide

Buffer 4 0.2 M borate buffer

Buffer 5 0.1 M ethanolamine

Buffer 6 10 mg/ml BSA solution containing 0.05% sodium azide

Buffer 7 Storage buffer containing 0.1% sodium azide

1. Place 0.5 ml of 2.5% suspension of polybeads into a 1.5 ml Eppendorf tube.
2. Add sufficient buffer 1 to fill tube and cap tightly.
3. Spin 5 minutes/ 12 Krpm.
4. Remove and discard supernatant.
5. Repeat steps 2-4.

To resuspend the pellet: fill tube halfway and cap; vortex; fill tube to capacity.

1. Resuspend pellet in buffer 2.
2. Spin 5 minutes/ 12 Krpm.
3. Remove and discard supernatant.
4. Repeat steps 6-8 twice.
5. Resuspend pellet in 0.625 ml of buffer 2.
6. Make 0.75 ml of a 2% carbodiimide by weighing out 15 mg of carbodiimide (bottle 3) and dissolving it in 0.75 ml buffer 2.

Carbodiimide solution should be prepared fresh and used within 15 minutes. The carbodiimide in bottle 3 should be protected from moisture. Immediately after using it, cap the bottle and put it at -20°C.

1. To the redispersed pellet, add 0.6 ml of the 2% carbodiimide solution dropwise while vortexing.
2. Mix for 3-4 hours at room temperature on an Eppendorf Thermomixer (1000 rpm).
3. Spin 5 minutes/ 12 Krpm and discard supernatant.
4. Resuspend pellet in buffer 2.
5. Spin 5 minutes/ 12 Krpm and discard supernatant.
6. Repeat steps 15 and 16 two more times to get rid of the unreacted carbodiimide.
7. Resuspend pellet in 1 ml buffer 4.
8. Add 300 µg of rabbit IgG.
9. Leave overnight at room temperature on Thermomixer, 850 rpm.
10. Spin 10 minutes/ 12 Krpm. Transfer supernatant completely to a small graduated cylinder or tube. Note the volume of the supernatant and save it for protein determination.

If this is done spectrophotometrically, make sure that the supernatant is free of turbidity (centrifuge supernatant for an additional 10 minutes). The value represents the amount of unbound antibody.

*I usually get a coupling efficiency of 60-80%.*

1. Resuspend pellet in 1.2 ml buffer 4. Add 50 µl of buffer 5 and mix for 30 minutes on Thermomixer, 850 rpm. This step blocks the unreacted sites on the beads.
2. Spin 10 minutes/ 12 Krpm and discard the supernatant.
3. Resuspend pellet in 1 ml buffer 6 containing additional heparin (0.4 mg/ml) and tRNA (0.1 mg/ml). Mix on Thermomixer for 30 minutes/ 850 rpm. This step serves to block nonspecific protein binding sites.
4. Spin 5 minutes/ 12 Krpm and discard supernatant.
5. Repeat steps 24 and 25 two more times.
6. Resuspend pellet in 0.5 ml buffer 7. Store **aliquot** at 4°C until needed. Use 4 aliquots for one affinity purification experiment.

**Ordering information:**

Polybead carboxylated 0.75-1 micron, Polysciences Inc, Cat No. 07759

Carbodiimide kit for carboxylated microparticles, Polysciences Inc, Cat No. 19539

Rabbit IgG, purified, Sigma, Cat No. I5006

tRNA from E. coli, Roche, Cat No. 10 109 550 001

Heparin sodium salt, Sigma, Cat No. H3400

# Affinity purification of ribosomes and associated RNAs

# Growth yeast cells in 50-100 ml YPD at 30°C to OD_600_ = 0.5.

1. Add cycloheximide to a final conc. of 0.1 mg/ml at 30°C for one minute, then harvest cells by vacuum filtration (HVPL type filters [0.45 µm]; Millipore).
2. Wash the cells directly on filter with10 ml ice-cold buffer A.

*Recipe for 10 ml*

Buffer A: 20 mM Tris-HCl (pH 8.0) 200 µl 1 M Tris-HCl (pH 8.0)

140 mM KCl 700 µl 2 M KCl

2 mM MgCl_2_ 20 µl 1 M MgCl_2_

1% Triton 500 µl 20% Triton

0.2 mg/ml heparin 100 µl 20 mg/ml stock

0.1 mg/ml cycloheximide 10 µl 100mg/ml stock in EtOH

8.48 ml RNase free H_2_O

1. Freeze filter in liquid nitrogen and store at -80°C.
2. Resuspend cells in 1.0 ml buffer B:

Buffer B: buffer A with 0.5 mM DTT

1 mM PMSF

0.5 µg/ml Leupeptin

0.8 µg/ml Pepstatin

100 U/ml RNase Out

20 U/ml DNase

Transfer suspension to a glass corex tube and add 2/3 vol. chilled glass beads (0.45-0.55 mm). 4x vortex hard for 20 sec and cool on ice in between for at least 90 sec.

1. Spin corex tubes at 2600g for 5 min/ 4°C. Transfer supernatant with a sterile pasteur pipette to 2 ml tube.
2. Spin 9.5 Krpm for 5 min/ 4°C in a table top centrifuge. Transfer to RNase-free 2 ml tube.
3. Spin 12 Krpm for 5 min/ 4°C. Transfer to RNase-free 2 ml tube.
4. Add buffer B to a final volume of 1 ml. This is the extract.

Store:

Remove 250 µl (10%) of the extract for further analysis, including:

- 20 µl for SDS-PAGE, combine with 20 µl 2x SDS-Laemmli buffer.

- 80 µl for total RNA isolation.

*Protein concentration ~ 6 mg/ml*.

1. Binding to polybeads (coupled to IgG):

Equilibrate beads in buffer A (wash the beads at least 3x with 1.5 ml buffer A for 10 min, spin at 12 Krpm).

Add beads to 0.75 ml extract (75%), put on Thermomixer for 2 h/ 850 rpm/ 4°C.

1. Spin beads down (2 min/ 6 Krpm/ 4 °C).

Store:

Supernatant, including:

- 20 µl SDS-PAGE, combine with 20 µl 2x SDS-Laemmli buffer.

1. Wash the beads 4x with ~2 ml buffer C for 15 min at 4°C on Thermomixer (750 rpm). Between the washes spin shortly (2 min) at 6 Krpm/ 4°C. Change the tube once during the procedure to prevent nonspecific bindings to the tube.

*Recipe for 10 ml*

Buffer C: 20 mM Tris-HCl (pH 8.0) 200 µl 1 M Tris-HCl 140 mM KCl 700 µl 2 M KCl

2 mM MgCl_2_ 20 µl 1 M MgCl_2_

5% glycerol 1 ml 50% glycerol

0.5 mM DTT 5 µl 1 M DTT

40 U/ml RNase out 400 Units

7.07 ml RNase free H_2_O

1. Resuspend beads in 690 µl buffer C

**Store:**

20 µl of beads for SDS-PAGE.

1. Add 60 µl of 10 U/ µl TEV-protease (final conc. of 0.8 U/µl). Final elution volume is 750 µl.
2. Incubate for 2 h/ 15°C on Thermomixer (750 rpm).
3. Spin 5 min/ 12 Krpm, remove supernatant (TEV eluate).

**Store:**

20 µl for SDS-PAGE.

Rest of TEV eluate for RNA isolation.
